# Extracellular loops of BtuB facilitate transport of vitamin B_12_ through the outer membrane of *E. coli*

**DOI:** 10.1371/journal.pcbi.1008024

**Published:** 2020-07-01

**Authors:** Tomasz Pieńko, Joanna Trylska

**Affiliations:** 1 Centre of New Technologies, University of Warsaw, Warsaw, Poland; 2 Department of Drug Chemistry, Faculty of Pharmacy with the Laboratory Medicine Division, Medical University of Warsaw, Warsaw, Poland; University of Uppsala, SWEDEN

## Abstract

Vitamin B_12_ (or cobalamin) is an enzymatic cofactor essential both for mammals and bacteria. However, cobalamin can be synthesized only by few microorganisms so most bacteria need to take it up from the environment through the TonB-dependent transport system. The first stage of cobalamin import to *E. coli* cells occurs through the outer-membrane receptor called BtuB. Vitamin B_12_ binds with high affinity to the extracellular side of the BtuB protein. BtuB forms a *β*-barrel with inner luminal domain and extracellular loops. To mechanically allow for cobalamin passage, the luminal domain needs to partially unfold with the help of the inner-membrane TonB protein. However, the mechanism of cobalamin permeation is unknown. Using all-atom molecular dynamics, we simulated the transport of cobalamin through the BtuB receptor embedded in an asymmetric and heterogeneous *E. coli* outer-membrane. To enhance conformational sampling of the BtuB loops, we developed the Gaussian force-simulated annealing method (GF-SA) and coupled it with umbrella sampling. We found that cobalamin needs to rotate in order to permeate through BtuB. We showed that the mobility of BtuB extracellular loops is crucial for cobalamin binding and transport and resembles an induced-fit mechanism. Loop mobility depends not only on the position of cobalamin but also on the extension of luminal domain. We provided atomistic details of cobalamin transport through the BtuB receptor showing the essential role of the mobility of BtuB extracellular loops. A similar TonB-dependent transport system is used also by many other compounds, such as haem and siderophores, and importantly, can be hijacked by natural antibiotics. Our work could have implications for future delivery of antibiotics to bacteria using this transport system.

## Author summary

Vitamin B_12_, also called cobalamin, is essential for mammalian and bacterial metabolism. However, only few microorganisms can synthesize vitamin B_12_ and in order to grow most bacteria need to retrieve it from the environment. Cobalamin uptake into *E. coli* first occurs through their outer-membrane protein called BtuB. The three-dimensional structure of BtuB has been resolved revealing that BtuB forms a *β*-barrel with inner luminal domain and extracellular loops. To mechanically enable cobalamin passage, luminal domain needs to partially unfold, but the detailed mechanism of cobalamin transport is still unknown. Using various molecular dynamics simulation techniques, we determined atomistic details of cobalamin transport through BtuB showing the mobility of BtuB extracellular loops during cobalamin binding and permeation. Our work implies future use of such transport systems to deliver antibiotics because a similar TonB-dependent transport system is used also by other compounds, such as haem and sucrose, and importantly, by natural antibiotics. Thus, our work can contribute to the development of delivery methods of antibiotics into bacteria.

## Introduction

The outer membrane of Gram-negative bacteria permits downhill diffusion of small hydrophilic molecules into the periplasmic space. However, bulky molecules (over ∼600 Da) or compounds present in the extracellular medium in scarce amounts, such as organometallic species, cannot permeate through porins, and their transport requires different routes. Since these compounds are fundamental for bacterial growth and viability, and largely determine the virulence of pathogens, bacteria developed specific and selective receptor-dependent active transport systems for these molecules. So far, this kind of system has been best characterized for vitamin B_12_, also called cobalamin (Cbl). Vitamin B_12_ is an enzymatic cofactor essential both for mammals and bacteria, but only few microorganisms can synthesize Cbl. Most bacteria including *Enterobacteriaceae*, *Bacillus subtilis* and group A *Streptococci* need to take up vitamin B_12_ from the environment.

In *E. coli*, transport of vitamin B_12_ across the outer membrane is facilitated by the transmembrane receptor protein BtuB [1, 2] that belongs to the family of TonB–dependent transporters (TBDTs). In addition to Cbl, TBDTs support the delivery of haem (recognized by the HasR and HemR outer-membrane receptors), ferric-siderophores (the FhuA, FhuE, FecA, and FepA receptors), sucrose (SuxA), and maltodextrin (MalA) [3, 4]. The TBDTs can be also hijacked by colicins [5], bacteriophages [6], and sideromycins [7] to enter the bacterial cells. Recently, we have shown that vitamin B_12_ delivers peptide nucleic acid and 2’O-methyl RNA oligomers into *E. coli* and *S*. Typhimurium cells [8–11]. Thus, vitamin B_12_ may be used as a carrier of various oligonucleotides to bacterial cells.

**Fig 1:**
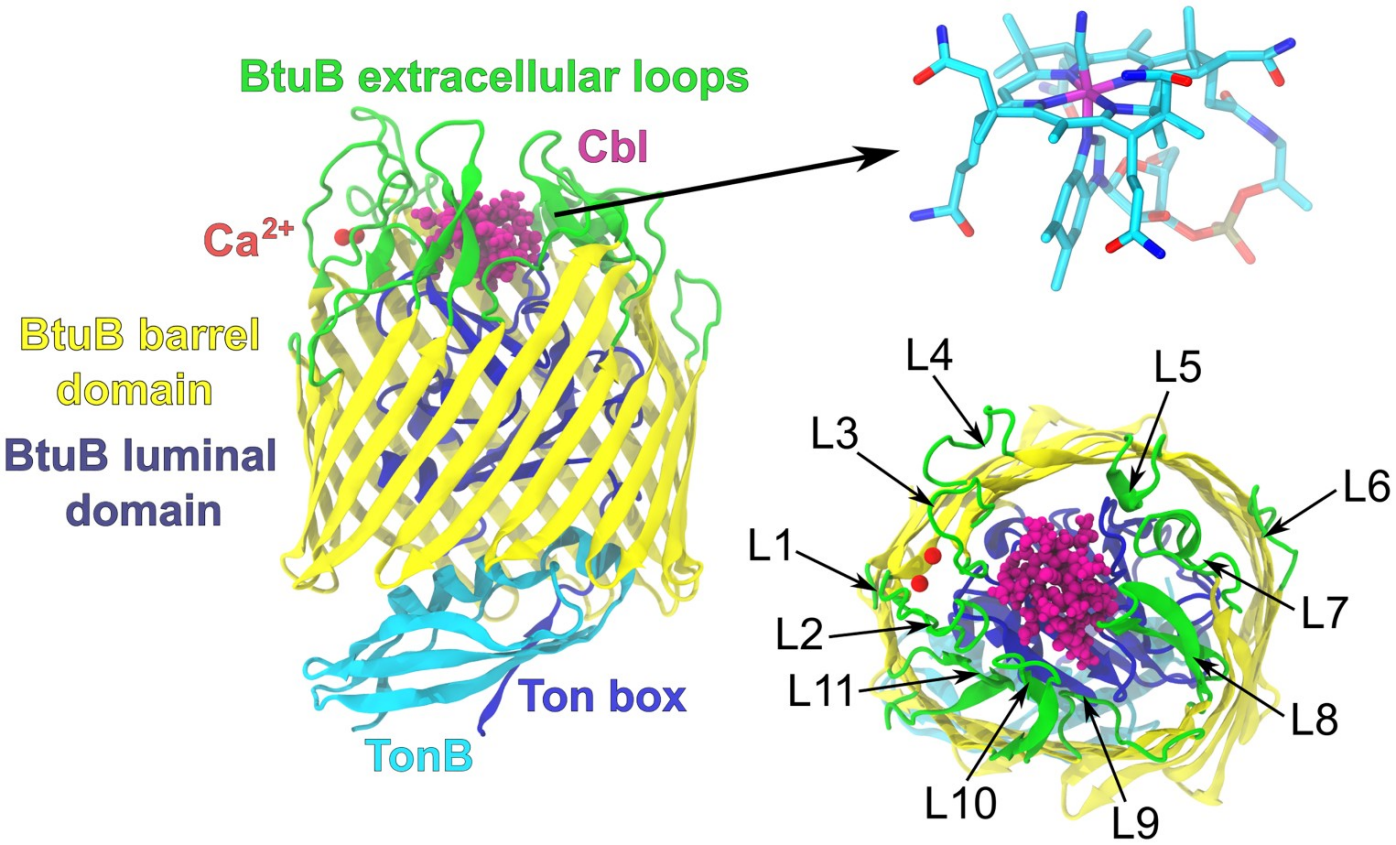
The BtuB protein. Left: The ribbon model of the BtuB structure in the complex with Cbl and C-terminal fragment of TonB [14]. BtuB extracellular loops are highlighted in green but in terms of topology they belong to the BtuB barrel domain. Right: The Cbl structure (only heavy atoms are shown) and numbering of BtuB extracellular loops in accord with [15].

The BtuB architecture is characteristic for TBDTs and organized in two domains—a 22-stranded *β*–barrel domain and N–terminal globular-like luminal domain (also known as a hatch or plug domain) that occludes the lumen of the BtuB barrel (Fig 1). The three-dimensional structure of BtuB has been resolved in the apo state (using detergent-based [12] and membrane-based crystallization [13]), in the apo state with bound calcium [12], in the holo state [12], and in the holo state in the complex with the C-terminal part of TonB [14].

The Cbl binding site is located on the two apices of the plug domain surrounded by the extracellular loops that partially sequester the ligand. The crystal structures of BtuB suggest that loops L2 through L5 play key roles in high-affinity binding of vitamin B_12_ [12, 16] (Fig 1). Also, partial deletions in loops L7, L8, L9, and L11 strongly decreased (but did not abrogate) Cbl binding and transport [17].

In proximity to vitamin B_12_ binding pocket, there are two calcium binding sites, contained in a cage formed by five aspartates [12, 16]. Intriguingly, for saturating calcium levels, the binding affinity of BtuB for Cbl is 5 nM, while at sub-saturating calcium concentrations, the Cbl affinity decreases 1000–fold [18–20]. The electron paramagnetic resonance (EPR) spectroscopy [21] and molecular dynamics (MD) simulations [22–24] suggested that calcium ions limit the conformational space sampled by loop L2 preventing its occlusion of the Cbl binding site.

The N-terminal periplasm–exposed region of the luminal domain conserved among the TBDTs [25], called Ton box, was shown crucial for Cbl transport [26–28] (Fig 1). In the apo state of BtuB, Ton box exists in equilibrium between the folded and unfolded states. Binding of vitamin B_12_ to BtuB shifts this equilibrium towards the disordered state [29–32]. As a result, Ton box extends approximately 20–30 Å into the periplasm, which may initiate its recognition by the TonB protein [33].

The TonB protein is anchored in the inner membrane by the N–terminal single transmembrane helix, which is connected to the extended proline-rich periplasmic domain. The C-terminal part of this TonB domain reaches out to the outer membrane [34], which enables it to form a non–covalent complex with Ton box of BtuB [14] (Fig 1). If not bound to the BtuB receptor, TonB exists mainly as a dimer, but during transport TonB is converted to a monomeric form to yield the high-affinity BtuB–TonB complex [35, 36]. TonB can allosterically affect the binding affinity of vitamin B_12_ for BtuB, however the directionality of this activity is still a matter of debate [21, 36].

Since the luminal domain of BtuB physically precludes free diffusion and passage of Cbl through BtuB, some conformational rearrangement of this plug, exposing a permeation pathway, has to be induced before TonB disengages from BtuB. The most probable mechanism of luminal domain rearrangement, upon its interaction with TonB, is the mechanical pulling hypothesis [25]. According to this model, TonB mediates a force that changes the plug conformation in a way that Cbl can permeate through BtuB and reach the periplasm. This force is believed to be generated by an electrochemical gradient of protons across the inner membrane utilized by ExbB and ExbD—TonB accessory inner-membrane proteins [37, 38]. However, how this proton-motive force is coupled to the outer-membrane BtuB transporter is still unknown. Single–molecule force spectroscopy studies have shown that the non–covalent interaction between the Ton box and C-terminal domain of TonB is durable under tension up to about 200 Å extension of the luminal domain [39]. According to steered molecular dynamics (SMD) simulations [39, 40], such extension, which corresponds to the unfolding of about 50 amino acids within the luminal domain, is sufficient to create a channel tailored for Cbl passage through the BtuB barrel. Bacterial growth assays suggested that such partial unfolding of the luminal domain precludes free diffusion of undesirable bulky molecules, such as antibiotics, into the cell thus narrowing the set of compounds allowed for passage and transport selectivity to Cbl [39].

One of the crucial issues that remains unanswered is the mechanism of passage of vitamin B_12_ through the BtuB itself. Since Cbl binds to the part of the luminal domain that unfolds upon TonB activity, it was speculated that Cbl could be pulled through BtuB. However, SMD simulations of unfolding of the luminal domain have shown that concurrent displacement of vitamin B_12_ towards the periplasm was negligible [39]. Thus, in this work, we investigate and describe complete permeation of Cbl through BtuB at atomistic level of detail using enhanced sampling molecular dynamics (MD) simulations, including umbrella sampling (US) simulations. We also identify the roles of BtuB extracellular loops in the transport of vitamin B_12_. To sample the conformational space of the loops in the outer-membrane environment, we developed the Gaussian force-simulated annealing method (GF-SA) and applied it to BtuB in different states of the transport cycle. Overall, we characterize the relation between the vitamin B_12_ passage, luminal domain unfolding and geometry of the extracellular loops, providing a whole atomistically-detailed picture of Cbl transport through BtuB.

## Methods

### System building

To build the BtuB model, the crystal structure of BtuB (PDB ID: 2GSK) [14] in the complex with cyanocobalamin from the OPM (Orientations of Proteins in Membranes) database [41] was used. Thus, in the text, for simplicity, we use the Cbl abbreviation for cyanocobalamin. The first four N-terminal amino acids missing in the BtuB structure were added, and the C-terminal fragment of TonB was removed. Protonation states of BtuB titratable groups were established and hydrogen atoms added with Propka 3.0 using the PDB2PQR server [42]. Cbl, water molecules and calcium ions were preserved as in the crystal structure. The FB15 AMBER force field [43] was used for BtuB. For vitamin B_12_, the AMBER bonded parameters developed by Marques et al [44] were applied. Non-bonded parameters for Cbl were set using General Amber Force Field 2 (GAFF2) and the partial charges were calculated according to the RESP procedure [45] using Gaussian 09 [46] and antechamber (AmberTools17).

The outer membrane of *E. coli* was built as an asymmetric heterogenous lipid bilayer. Lipids were assembled around BtuB using CellMicrocosmos Membrane Editor 2.2 [47]. The external leaflet of the membrane was made of 29 lipopolysaccharide (LPS) molecules to mimic the K-12 strain of *E. coli*. This LPS possesses the membrane-forming lipid A, as well as the inner and outer-core oligosaccharides but does not have the O antigen. The interior leaflet of the membrane was composed of six kinds of lipids: POPE, PMPE, PMPG, PSPG, QMPE, and OSPE with the ratio of 8:31:8:8:8:6 [48]. Bonded and non-bonded parameters for lipids were determined using GAFF2 with the partial atomic charges calculated according to the RESP procedure [45] using Gaussian 09 [46] and antechamber (AmberTools17).

**Fig 2:**
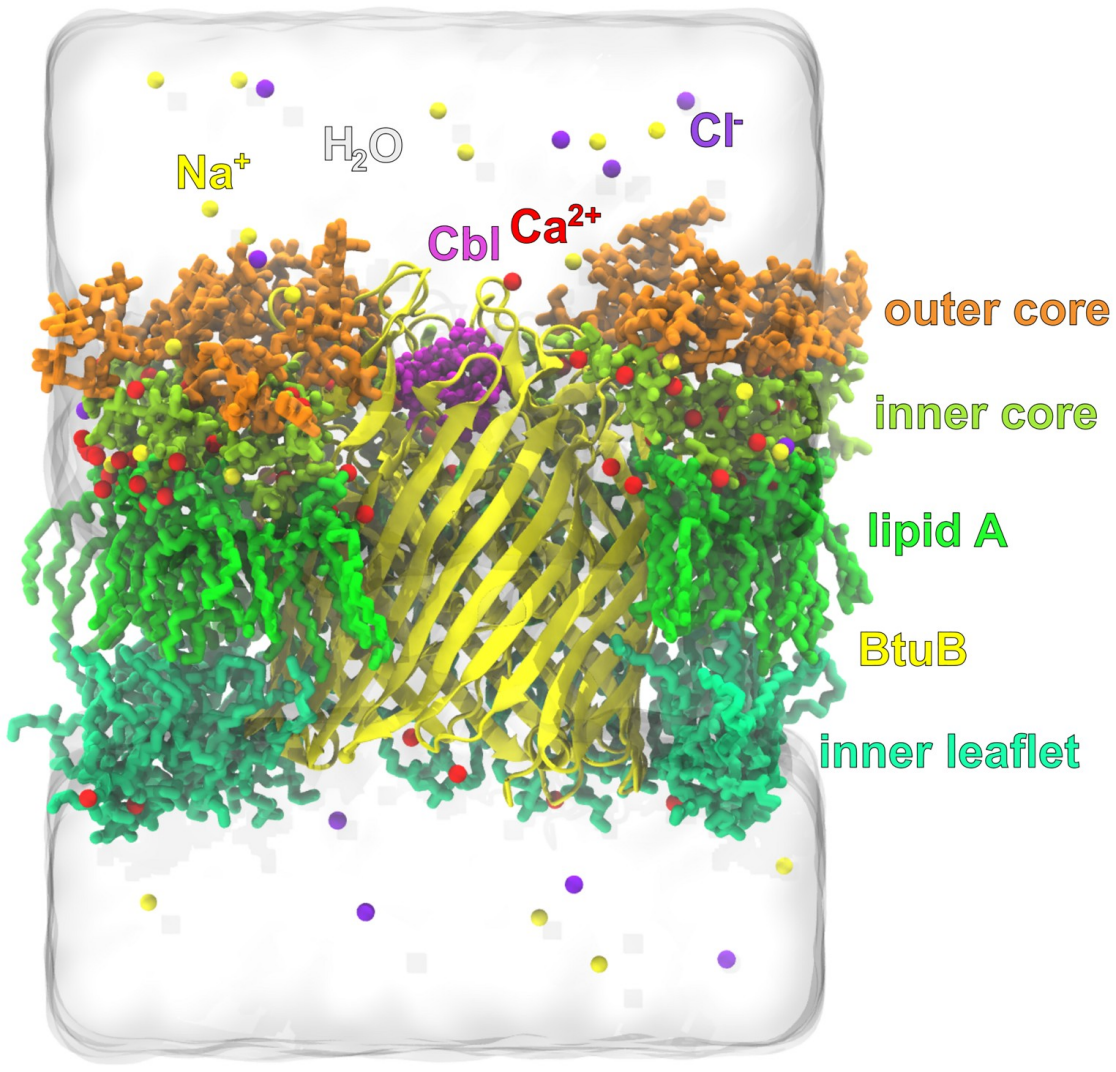
The simulated system. The full-atomistic model of BtuB embedded in the heterogeneous and asymmetric outer membrane of *E. coli* K-12 in the environment of Ca^2+^, Na^+^, Cl^−^ ions and water.

Using xleap (AmberTools17) [49] and Visual Molecular Dynamics (VMD) [50], 20 Å and 280 Å layers of water molecules (in the z-coordinate) were added over and below the membrane, respectively. The initial dimensions of system were 95 Å x 95 Å x 380 Å. The negative potential of LPS was neutralized with calcium ions, while the negative charge of the lower-leaflet lipids was balanced by adding sodium ions. In addition, ionic strength of 100 mM of NaCl was applied. The TIP3P-FB water model [51] was used as the one corresponding with the FB15 AMBER force field parameters for the protein. For the sodium and chloride ions, parameters developed by Joung and Cheatham [52], and for the calcium ions, by Li and Merz [53], were applied. The entire membrane-BtuB receptor system with Cbl consisted of about 296,000 atoms (Fig 2).

To confirm the correctness of the prepared asymmetric membrane model, we measured the area per lipid and membrane hydrophobic thickness. The area per lipid was computed using Voronoi tessellation Monte Carlo integration algorithm implemented in the VTMC program [54]. Membrane thickness was calculated as the difference between the average z-coordinate of C2 atoms of the acyl chains 4 and 6 and C4 atoms of the acyl chains 1, 2, 3, and 5 of LPS and average z-coordinate of C2 atoms of acyl tails of all of lower-leaflet lipids [55]. The average area per lipid of LPS is 151 Å^2^, which agrees with the experimental value of 156 Å^2^ [56]. The average membrane thickness is 24.05±0.07 Å, which matches the average hydrophobic thickness of all known outer-membrane proteins (∼24 Å) [57].

### General molecular dynamics simulations protocol

Initially, the systems were energy minimized (under harmonic restraints of 10 kcal/mol/Å^2^ set on non-hydrogen atoms) with the steepest descent method (5000 steps) followed by the conjugate gradient method (3000 steps) using the sander program [49]. Subsequent phases were carried out using NAMD 2.12 [58]. After minimization, the solute coordinates were fixed, and the system was gradually heated from 10 to 310 K, for 125 ps in 10 K increments, in the NpT ensemble, with a constant pressure of 1 atm controlled using the Langevin Piston method. The integration time step was set to 0.5 fs. Next, the water molecules and ions were equilibrated at 310 K for 2.5 ns. Further, the solute was restrained with a harmonic constant of 10 kcal/mol/Å^2^ imposed on heavy atoms, and the whole system was gradually thermalized from 10 to 310 K, in 10 K increments of 50 ps length. During equilibration, the restraints were removed over ten rounds of 50 ps each. Next, the integration time step was gradually increased from 0.5 fs to 2 fs, in 0.25 fs increments of 1 ns length, and the whole system was simulated for 400 ns. Periodic boundary conditions and Particle Mesh Ewald method with a grid spacing of 1.0 Å were used. The bonds involving hydrogens within non-water and water molecules were constrained using RATTLE [59] and SETTLE [60] algorithms, respectively. The cutoff for short-range non-bonded interactions was set to 12 Å, with a switching distance of 10 Å. Data were saved every 5 ps.

### Steered molecular dynamics

To unfold the BtuB luminal domain in a controlled manner, we used constant-velocity steered molecular dynamics (SMD) [61, 62]. The center of mass (COM) of the heavy atoms of the first N–terminal residue was pulled along the z-axis with a constant velocity of 1.0 Å/ns and a force constant of 1 kcal/mol/Å^2^. The force vector was [0, 0, -1]. The COM of the C_*α*_ atoms of the lower part of the BtuB barrel domain was harmonically restrained, with a force constant of 100 kcal/mol/Å^2^, to prevent the protein-membrane system from drifting along the z-axis upon the BtuB luminal domain pulling.

Our preliminary simulations have shown that the release of vitamin B_12_ from the BtuB protein requires additional force applied to vitamin B_12_ (see Results and discussion). We observed that unfolding of the luminal domain during constant-velocity SMD does not result in vitamin B_12_ transport even though the lumen becomes open and there is enough space for Cbl passage. This finding corroborates the studies of Hickman et al. [39]. However, we found that the release of vitamin B_12_ is precluded because it is strongly held by the BtuB extracellular loops. Therefore, to allow for vitamin B_12_ passage through BtuB, we applied external constant force on the COM of Cbl with a vector [0, 0, -x], where x was equal to 0, 5, 8, 10, 15, 20 kcal/mol/Å.

### Umbrella sampling

To compute the potential of mean force (PMF) of the association and permeation of vitamin B_12_ through BtuB, we applied the umbrella sampling (US) methodology [63]. The reaction coordinate was the distance projected onto a normal to the membrane (z-axis) between the COM of Cbl and dummy atom fixed in the center of the Cbl binding pocket. To make the position of the binding pocket comparable between different BtuB systems used in this study, we defined it as the average location of the COM of Cbl in the holo state of BtuB with luminal domain folded (the S2 state described in Results and discussion). The initial association and permeation pathway of vitamin B_12_ through BtuB was obtained using SMD. Cbl was pulled from the binding pocket (z = 0) with a constant velocity of 1.0 Å/ns and a force constant of 1 kcal/mol/Å^2^ along the z-axis. The force vector was [0, 0, 1] and [0, 0, -1] for association and permeation, respectively. Concurrently, to maintain the plug domain extension of 197 Å, the COM of the N–terminus of the luminal domain was harmonically restrained with a force constant of 100 kcal/mol/Å^2^. The US windows were spaced every 0.5 Å in the range of -30–70 Å of the reaction coordinate, and the force constant was set to 50 kcal/mol/Å^2^. Simulations in each US window lasted 37.5 ns, but first 2.5 ns were treated as an equilibration of the system properties, such as density, total energy and its contributions, as well as RMSD of BtuB–Cbl, thus were discarded from analysis. The PMF was computed using the Weighted Histogram Analysis Method (WHAM) [64, 65] with the WHAM program [66]. The convergence of PMF was monitored every 0.2 ns by measuring the root-mean-square deviation (RMSD) of PMF calculated from cumulative data. The reference for PMF RMSD calculations was the last PMF (computed based on all of the collected data).

### Gaussian force-simulated annealing method

To optimize the structure and enhance conformational sampling of the extracellular loops in different BtuB states (described in Results and discussion), we developed a Gaussian force-simulated annealing (GF-SA) method. During a typical simulated annealing protocol, a whole system is heated and, subsequently, slowly cooled down to find its global energy minimum. In our approach, the variable in the simulated annealing is not the temperature of the system but external Gaussian forces imposed locally on the BtuB loops.

The idea is to augment the rotation of chemical groups around the rotatable bonds in the protein backbone and side-chains. In the backbone, there are two freely rotatable bonds: C_*α*_-C and N-C_*α*_. The CO-NH moiety that connects these bonds is a planar rigid structure that prefers the *trans* position. Therefore, the conformational variety within the backbone arises from the relative positions of the CO-NH groups. In turn, the conformational variability of side-chains dominantly comes from the rotation of the residues around the C_*α*_-C_*β*_ bond. Thus, to enhance protein dynamics within the backbone and side-chains, we applied external forces to the atoms neighboring with the atoms involved in rotatable bonds (mentioned above). In the protein backbone, this applies to the O atom, whereas in side-chains the atom types depend on the residue (e.g., C_*γ*_, O_*γ*_, etc.).

The forces imposed on the atoms were calculated as normalized vectors. The vectors were normal to the planes formed by the following angles: C_*α*_-C-O in the backbone and C_*α*_-C_*β*_-X in side-chains, where X is C_*γ*_, O_*γ*_, etc. The forces were multiplied by a pseudo-random number from the Gaussian distribution with the zero average and fixed standard deviation (see below). The C++ 11 Standard Library *random* class was used to generate the random numbers. In addition, to make random numbers uniformly distributed between the average and standard deviation, we applied the Knuth-b generator. Then, we used the Box-Muller method to transform these numbers to normally distributed. Each Gaussian number was produced using a new seed used by the generator. The seed was assigned nondeterministically by using the random device function. We found this procedure more effective in maintaining the normal distribution of pseudo-random numbers than using only one initial seed followed by the update of the generator state.

The external forces were added every second step of MD simulation and were imposed on the following residues: 177-197 (loop L2), 227-241 (loop L3), 276-289 (loop L4), 323-333 (loop L5), 395-412 (loop L7), 442-458 (loop L8), 487-499 (loop L9), 525-542 (loop L10), 569-584 (loop L11). The rigid and short loops 1 and 6, as well as prolines, were intact. The affected residues were chosen based on their structure and root-mean-square fluctuations during conventional MD simulations of the holo state of BtuB.

In the GF-SA procedure, the standard deviation of the normally distributed pseudo-numbers, which determines how strongly the dynamics of the loops is enhanced, is changed over the simulation time. The standard deviation *s* was calculated according to the function:

$$s=-\frac{a}{b^{n}}*x^{n}+a;x\in <-b,b>,n=2,4,6,8\ldots$$ (1)

where *a* is a maximal standard deviation, *b* is an integer determining the number of stages during heating or cooling, *n* is an even integer controlling the gradient of the standard deviation. We set *a* to 35 kcal/mol/Å, *n* to 2, and *b* to 100. Heating was conducted in 100 stages *x* ∈ < −100, 0) for 2000 MD steps each. Then, the standard deviation was maintained in its maximum (*x* = 0) for 250000 MD steps. Subsequently, cooling was carried out in 100 stages *x* ∈ (0, 100 > for 30000 MD steps each. In the systems simulated with vitamin B_12_, during heating and cooling, the ligand was harmonically restrained with a force constant of 10 kcal/mol/Å^2^. The restraint on vitamin B_12_ was released for subsequent 50000 MD steps followed by conventional MD simulation (with no Gaussian forces). Then, the whole procedure, GF-SA-MD, was repeated for a number of cycles. To prevent the protein-membrane system from artificial drifting upon the external Gaussian forces imposed on the BtuB loops, the COM of the C_*α*_ atoms of the lower part of the BtuB barrel domain was restrained harmonically with a force constant of 100 kcal/mol/Å^2^.

The GF-SA method was implemented in NAMD using the TclForces module. The general code of the method was written in Tcl, however all mathematically robust operations were written in C++, and as an extension wrapped into the Tcl script. The wrapper code was generated using the Swig 3.0 program [67].

### Data analysis

Trajectories were analyzed with the cpptraj program (AmberTools17) and VMD 1.9.3 using in-house Tcl and C++ scripts. VMD was also used for visualization. Plots were generated with Gnuplot (v5.2). The criteria for hydrogen bonds were set to 3.5 Å between the donor and acceptor, and no less than 135 degrees angle between donor–hydrogen–acceptor. To detect the contacts, the distance of 3.5 Å was used as the sole criterion. Descriptive statistics calculations were performed using in-house C++ scripts. Data sets underwent the Kolmogorov-Smirnov test to check the normality of the distribution. The data series with a not normal distribution were characterized by median, interquartile range (Q1-Q3), and lower–upper extremes range. In GF-SA-MD simulations, only data collected from the conventional MD were analyzed.

## Results and discussion

### Conventional MD does not properly describe the translocation of vitamin B_12_ through BtuB

**Fig 3:**
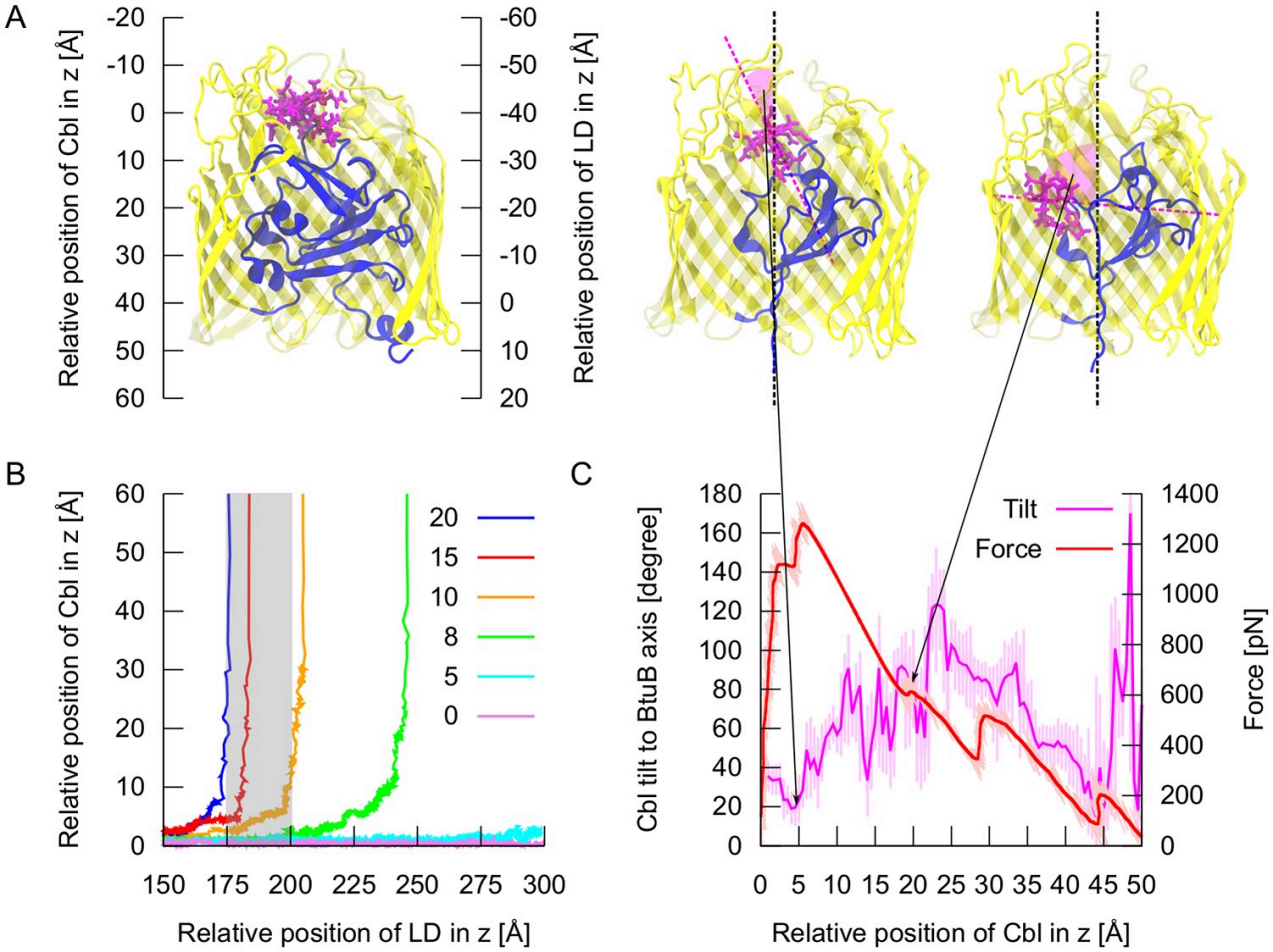
The release of Cbl from BtuB using conventional SMD simulations. **A**: The coordinate system adopted to follow the position of vitamin B_12_ and luminal domain (LD) along the z-axis. **B**: The release of Cbl upon external force (0–20 kcal/mol) at different extensions of the BtuB luminal domain. The gray area indicates the window in which the release of Cbl may occur, corroborating the AFM data [39]. **C**: The magnitude of the steering force necessary to release Cbl at 197 Å of the luminal domain extension (the extended part of luminal domain is not shown for the clarity of the figure). Rotation of vitamin B_12_ relative to the main axis of the protein during SMD simulation of Cbl permeation through BtuB.

So far, only Hickman et al. [39] performed SMD simulations of unfolding of the BtuB luminal domain in the presence of Cbl, to find a link with their AFM data. In the beginning, we performed analogous SMD simulations, and, similarly, we did not observe any substantial motion of vitamin B_12_ towards the periplasmic space (S1 Fig). Cbl was held by the extracellular loops and did not follow the luminal domain upon its unfolding. Thus, to enhance Cbl movement, simultaneously with unfolding of the luminal domain, we imposed a constant force on the Cbl center of mass, directed toward the periplasm along the normal to the membrane (see S1 Video). Depending on the magnitude of this force (Fig 3B), we observed the release of vitamin B_12_ at different extensions of the luminal domain. We noted that the earliest full permeation of Cbl was mechanically feasible once the luminal domain was extended to at least 175 Å and upon the external force of 20 kcal/mol/Å. We also found that in the first stage of passage (z = 0–24 Å), vitamin B_12_ rotates along the main axis of BtuB in such a way that the Cbl corrin ring becomes oriented parallel to this axis (Fig 3C). This indicates that plain translation of Cbl through the BtuB barrel would be mechanically ineffective.

However, SMD provides only a qualitative picture of vitamin B_12_ permeation. To obtain quantitative data, namely the potential of mean force (PMF) of Cbl passage through BtuB, we applied US. Since Cbl transport is concomitant with unfolding of the BtuB luminal domain, the complete PMF depends on both the position of vitamin B_12_ relative to the BtuB barrel and extension of the luminal domain. Thus, the PMF is at least two-dimensional. However, due to huge computational cost of 2D US simulations, we simplified this problem to 1D US calculations.

Hickman et al. [39] have shown by AFM that the interaction between Ton box and TonB is stable up to about 200 Å extension of the luminal domain. Therefore, the window for releasing vitamin B_12_ should span the 175–200 Å extension of this domain. During the conventional SMD simulation of luminal domain unfolding, we observed that the highest steering force peaks occur at 160 Å and 215 Å of the luminal domain extension (S1 Fig). Thus, the partially unfolded BtuB becomes most stable while releasing vitamin B_12_. We also identified three local minima of the SMD force within the permeation window, at the 175 Å, 185 Å, and 197 Å extension. The latter, most stable extension, is the maximal up to which TonB can “pull” the luminal domain without breaking of their interactions [39], and is also sufficient to make space for Cbl passage within BtuB. Therefore, we assumed that Cbl passes through BtuB when the luminal domain fluctuates around its 197 Å extension point.

Subsequently, we performed 1D US calculations of vitamin B_12_ permeation under the restrained position of the luminal domain N–terminus at 197 Å extension, which allows for Cbl passage from the mechanical point of view. However, after 35 ns of US simulations, we obtained a highly unstable PMF (S2 Fig), especially in the first stage of Cbl transport, at 0 < z < 30 Å. Notably, during this phase, vitamin B_12_ interacts strongly with the BtuB loops requiring steering force for its release (Fig 3C). The loops tightly sequester Cbl at the binding site, and remain stable throughout the simulations. In addition, such rigid and compact configuration of the loops hampers the rotation of vitamin B_12_ that is necessary for its permeation (Fig 3C). Therefore, we concluded that the time scale of conventional MD is insufficient to extensively sample the conformational space of the BtuB extracellular loops.

Furthermore, due to crystallization conditions, the conformations of the loops resolved based on the BtuB crystal may be different than in physiologic state. Numerous studies on BtuB, such as EPR spectroscopy and site-directed spin labeling, demonstrated that either the solutes or osmolytes used in the crystallization buffer alter the conformational sampling of the solvent-exposed protein segments, such as Ton box and extracellular loops, favoring more compact, ordered, and less hydrated structures [32, 68–72]. A similar energy contribution could arise from the crystal lattice [32]. It was also proven that the detergent-based or membrane-based crystallization method affected the ordering of the BtuB extracellular loops and Ton box conformation [12, 13]. Additionally, the most recent EPR spectroscopy measurements have shown that the BtuB loops sample a much broader conformational space than revealed by the crystal structures [21]. According to Sikora et al. [21], in the crystals, BtuB extracellular loops are conformationally trapped, representing one of the more compact structures among those sampled in the outer membrane. Thus, efficient conformational sampling of the loops is a must.

### Conformational changes of the BtuB loops during the vitamin B_12_ transport

**Fig 4:**
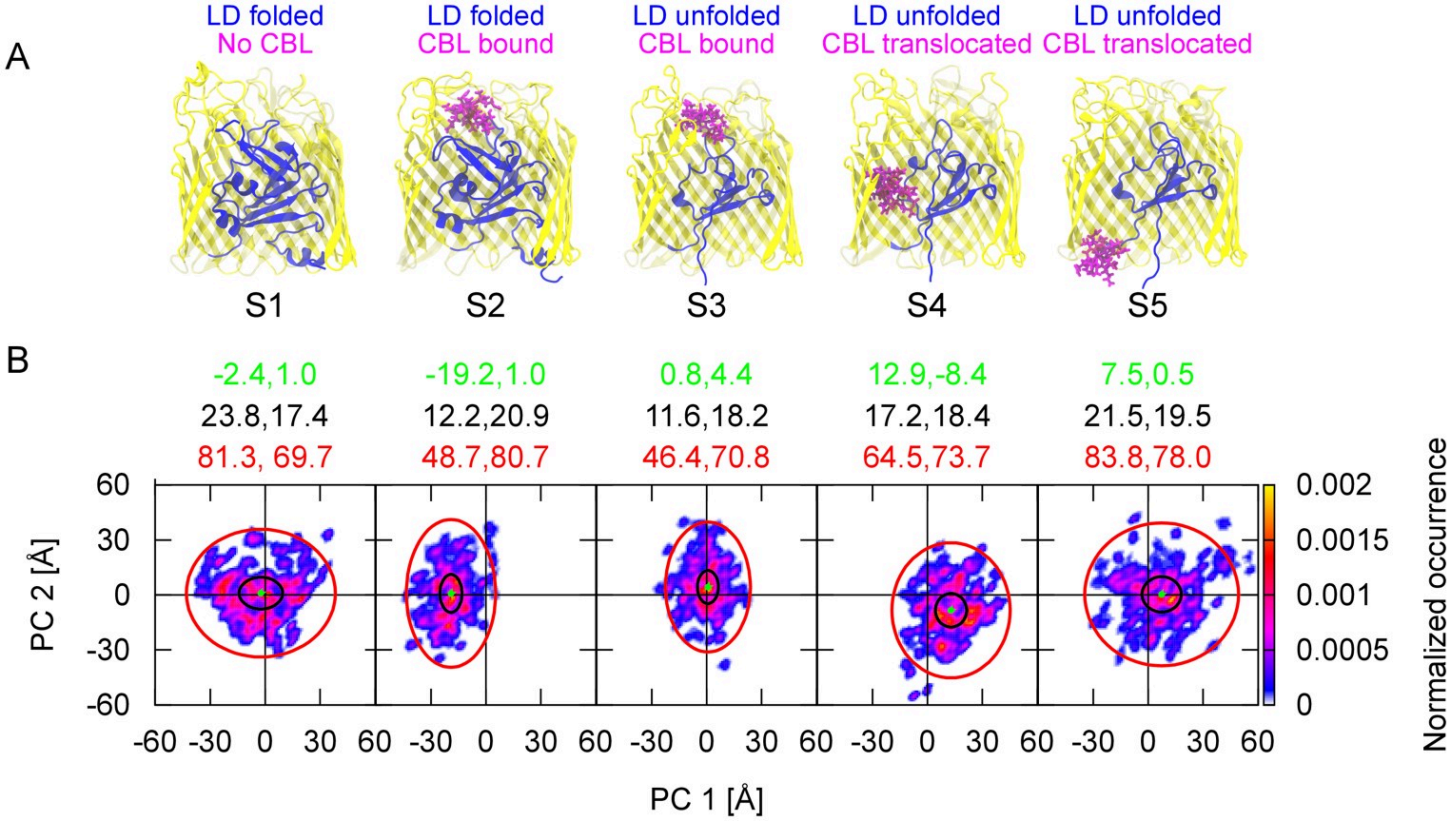
BtuB conformational states and mobility of the loops. **A**: The states of BtuB investigated in this study: the apo state (S1), the holo state with the luminal domain folded (S2), the holo state with the luminal domain partially unfolded (S3), the holo state with the luminal domain partially unfolded and Cbl shifted towards the periplasm (25 Å in S4 and 50 Å in S5). **B**: The distribution of the first two principal components (PCs) of the BtuB barrel domain in different states of BtuB (S1–S5). For PC 1 and PC 2, the medians (in green), interquartile ranges (in black), and lower–upper extremes (in red) are also shown.

To investigate the mobility of the BtuB extracellular loops and their role in Cbl transport, we developed an enhanced sampling Gaussian force-simulated annealing method, GF-SA, and coupled it with conventional MD (for details see Methods and S2 Video). In the GF-SA-MD simulations, we considered different states of the BtuB protein corresponding to different stages of the transport cycle (Fig 4A) (see S3 Video). Their initial structures were obtained using SMD simulations described above. These states were: the apo state (S1), the holo state with the luminal domain folded (S2), the holo state with the luminal domain partially unfolded (S3), and the holo states with the luminal domain partially unfolded and Cbl shifted towards the periplasm (S4 and S5). Partial unfolding corresponds to the extension of the luminal domain at 197 Å in the z-axis with respect to the initial position. This extension was maintained by applying a harmonic restraint to the N–terminus of the luminal domain with the force constant of 50 kcal/mol/Å^2^. In S4 and S5, vitamin B_12_ was positioned, respectively, 25 Å and 50 Å in the z-axis towards the periplasm (for the axis describing the positioning of vitamin B_12_ and luminal domain see Fig 3A). For each system, we performed 10 independent simulations, each consisting of 15 consecutive cycles (see S3 Video). Each cycle comprised 7 ns of GF-SA followed by 7 ns of conventional MD.

To assess the conformational sampling of the loops in the GF-SA-MD simulations compared to conventional MD on the microsecond time scale, we measured the distances between several loop residues in state S2, analogously to the EPR spectroscopy measurements of Sikora et al. [21]. Direct comparisons between the distance distributions obtained by EPR and GF-SA-MD are error-prone because the EPR were measured for spin-labeled residues, while the GF-SA-MD for natural, non-labeled ones. However, in GF-SA-MD for all distances, the distance distributions were substantially more spread, suggesting enhanced sampling of loop conformations as compared to conventional MD (S3 Fig). Therefore, we argue that GF-SA-MD may provide a more realistic picture of mobility of the BtuB loops than conventional MD.

To detect the differences in the conformations of the loops in different BtuB states (Fig 4A), on combined MD trajectories, we performed Principal Component Analysis (PCA) of the BtuB barrel domain C_*α*_ atoms. The first 50 eigenvectors contributed to over 80% of the total mean square fluctuations. Respective trajectories were projected onto the principal components (PCs) and showed largest variations in the two lowest frequency modes PC 1 and PC 2 (Fig 4B). We found that these PC modes correspond to the opening (PC 1 < 0 or PC 2 > 0) and closing (PC 1 > 0 or PC 2 < 0) motions of BtuB (see S4 and S5 Videos), which mainly involve loops L2, L3, L7, L8, L10, and L11 (S4 Fig).

**Fig 5:**
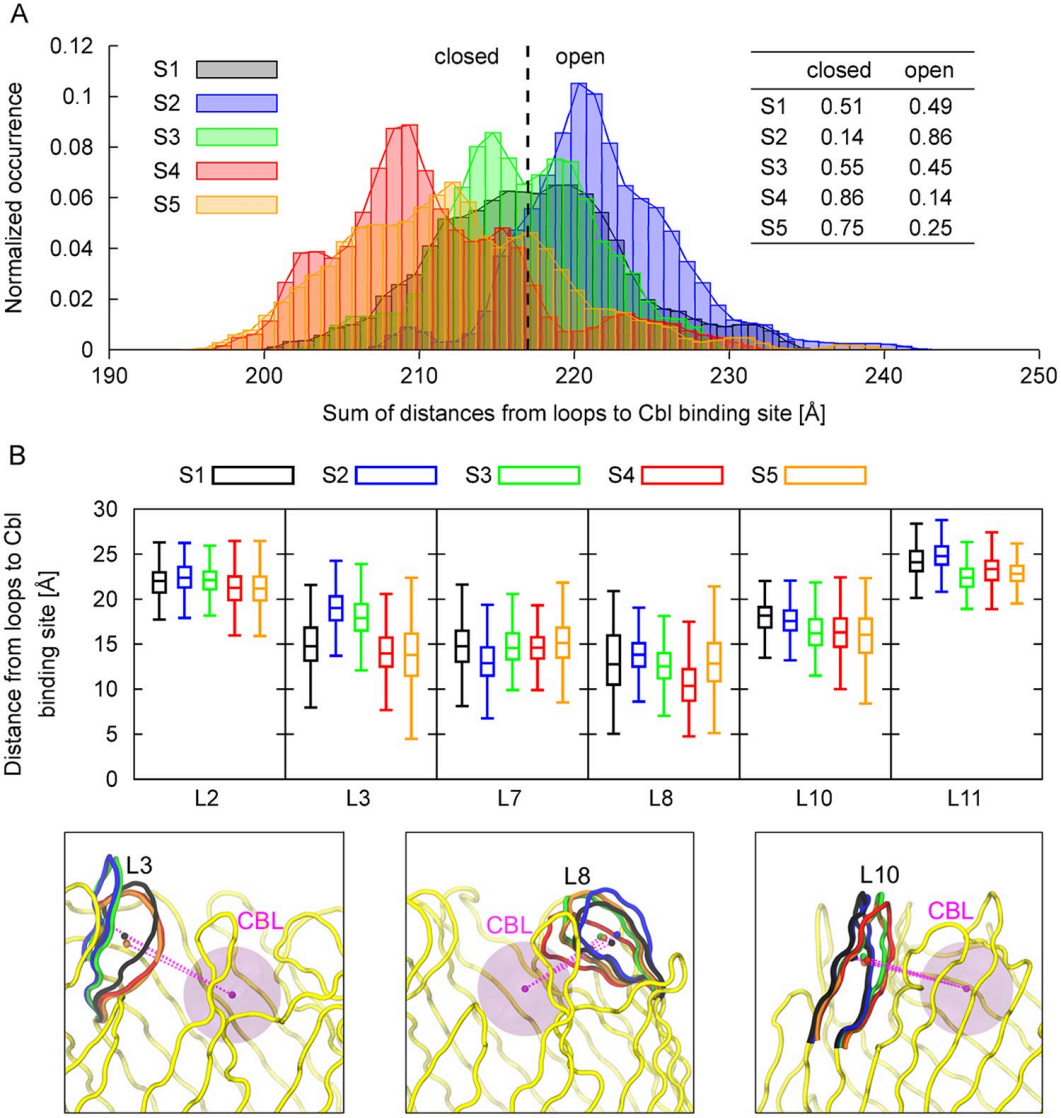
Contributions of loops to closing–opening motions in different BtuB states. **A**: The distributions of the sum of distances between the COMs of BtuB loops and Cbl binding site in different BtuB states as a measure of the probability of the closed and open BtuB geometries. The dashed line indicates the sum of distances of 217 Å considered a boundary between the closed (<217 Å) and open (>217 Å) conformations of the loops. **B**: The medians, interquartile ranges, and lower–upper extremes of the distance distributions between the COMs of the extracellular loops and Cbl binding site shown only for the loops that contribute the most to the closing–opening motions of BtuB. Lower insets show the distances (pink dashed lines) between the COM of Cbl binding pocket and COM of the average conformations of the selected loops. Loops are colored according to the BtuB states shown in Fig 4A.

To estimate the probability of the closed and open loop geometries in different BtuB states, we summed the distances between the center of masses of individual loops and the center of mass of Cbl binding site (Fig 5A). The boundary between the closed and open BtuB geometries was defined as the conformation of the BtuB barrel domain (Fig 1) averaged over all trajectories (for which both PC 1 and PC 2 equal 0 Å). We also analyzed the distance distributions of particular loops to Cbl binding pocket to quantify their individual contributions to the closing–opening motions in the different BtuB states (Fig 5B, S1 Table, S5 Fig).

In the apo state of BtuB (S1), as shown in Fig 4B, both PC 1 and PC 2 are widely and almost symmetrically distributed. This agrees with the ratio of the closed to open loop geometries of about 0.5 (Fig 5A). This also suggests an equilibrium between the closing and opening movements of the loops, with BtuB preference to adopt a neutral conformation (in which both PC 1 and PC 2 are positioned around 0 Å). The largest share in these motions comes from the loops L8, L3, and L7 (Fig 5B).

Binding of Cbl (S2) restricts the distribution of PC 1 and shifts it towards negative values (Fig 4B). Thus, in S2, the open conformation of BtuB loops is more occupied (0.86) than the closed one (0.14) (Fig 5A). The reason lies in steric hindrance caused by bound Cbl, which makes the loops, especially L3 and L8, protrude outside the Cbl binding site and lowers their mobility. In contrast, loop L7 prefers to move closer to the binding pocket upon Cbl binding (Fig 5B).

In state S3, when the luminal domain gets partially unfolded, PC 1 and PC 2 are again nearly symmetrically distributed (as in S1). However, the range of PC 1 is narrower than is S1 but similar as in S2, suggesting lower amplitude of loop motions in the direction of PC 1 (Fig 4B). In state S3, the probability of loop closing motions increases to 0.55 (Fig 5A) because loops L3, L8, L10, and L11 approach the binding site more often (Fig 5B). This implies that partial unfolding of the luminal domain may serve as a trigger to initiate the closure of the loops during Cbl transport.

In S4, after partial permeation of Cbl, the range of PC 1 shifts towards positive and of PC 2 towards negative values (Fig 4B), and the closed–open loop occurrence becomes opposite to the one of S2 (Fig 5A). This signifies that in S4 the loops prefer an even more closed geometry (0.86) than in S3. The closure of the BtuB barrel is mainly contributed by loops L3 and L8. Besides, in S4, the PC 1 range widens as compared to S3, indicating increased loop mobility. This is mainly observed for loops L2 and L10 (Fig 5B).

When vitamin B_12_ is fully permeated (state S5), the distribution of PC 1 and PC 2 resembles a mix of that in S1 and S4 (Fig 4B). As in S1, both PCs span a wide range of values suggesting large loop mobility. Indeed, most of the loops in S5, including L3, L7, L8, and L10, sample wide conformational space (Fig 5B). However, while PC 2 is symmetrically distributed (like in S1), PC 1 samples more positive values (like in S4). This is because, in S5, loops L2, L3, L10, and L11 approach the Cbl binding site more frequently than in S1 (Fig 5B). Nevertheless, in S5, the closing motions of the loops are less frequent than in S4, mainly due to moving of loop L8 outside the binding pocket. Overall, in states S3–S5, during Cbl permeation with luminal domain partially unfolded, the loops prefer the closed conformations.

### Non-specific interactions govern the mobility of BtuB loops

**Fig 6:**
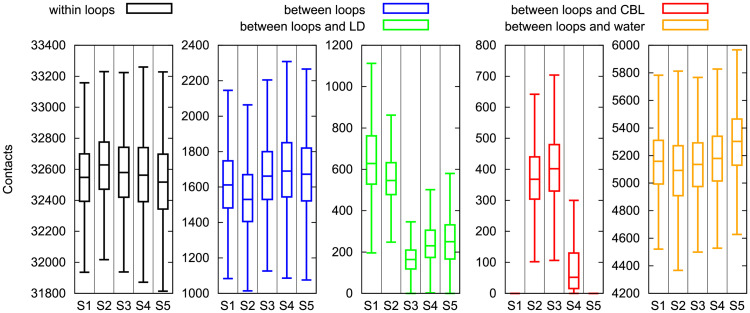
Loop, luminal domain and Cbl mutual interactions. The contacts within and between the loops, between the loops and luminal domain (LD), between the loops and Cbl, and between the loops and water in different BtuB states. For the definition of BtuB states see Fig 4A. The median, interquartile range, and the lower–upper extremes of each data series are provided.

To explain the conformational changes of the extracellular loops in various states of BtuB, we investigated their contacts and energetic properties. The number of contacts *within* the loops varies in the BtuB states and is maximal in state S2 upon Cbl binding (Fig 6). Then, while moving from state S2 to S5, the intra-loop contacts gradually decrease, with a change of over 100 in the median (Fig 6 and S2 Table). Since the number of hydrogen bonds formed *within* the loops is similar in these states (S2 Table, S6 Fig), the changes in the intra-loop contacts can be associated with non-specific interactions.

The number of contacts *between* the loops also depends on the BtuB state and increases during Cbl permeation (states S3–S5) (Fig 6, S7 Fig). Again, the hydrogen bonds *between* the loops practically do not change between the BtuB states (S6 Fig). This suggests that most inter-loop contacts during Cbl transport are non-specific, which is also reflected in the energetic properties of these loops.

Naturally, with more contacts between the loops, the van der Waals energy of their interactions becomes more negative (S7 Fig). However, despite the fact that for most loop pairs (except pairs including L9 and L10), the electrostatic interaction energy becomes more repulsive from state S1 to S5, it is balanced by the stabilizing van der Waals forces.

Not surprisingly, the number of contacts *between* the loops is the lowest in state S2 because the loops interact also with Cbl and folded luminal domain. Furthermore, in S2 most loops maximize their intra-loop connections (Fig 6). However, one could ask why the number of contacts *between* the loops is on average lower in S1 than in S5 (Fig 6) since in both states, the loops lack interactions with vitamin B_12_. This occurs because in S1 the number of contacts between the loops and luminal domain is the highest (Fig 6, S3 Table). Thus, it seems that in the apo state of BtuB (S1), the loops, mainly L2, L3, L7, L8, L9, L10 and L11, prefer to interact with the luminal domain rather than with themselves. On the contrary, in S5, after Cbl translocation, many of the interactions of the loops with luminal domain disappear due to its partial unfolding (S8 Fig), and this is compensated by increasing the inter-loop connections.

Analogous phenomenon occurs while moving from state S2 to S3. Interestingly, the intensified contacts *between* the loops in S3 are accompanied by the increased interactions with Cbl (Fig 6, S9 Fig).

The contacts between the loops and luminal domain gradually increase during permeation of vitamin B_12_, i.e., between state S3 and S5. When the number of contacts between the loops and Cbl decreases, the loops compensate it by interacting with themselves and luminal domain. Most of these contacts are non-specific, because the number of direct and water-mediated hydrogen bonds of the loops with luminal domain remains almost unaffected (S3 Table). In addition, for most loops their electrostatic interaction energy with the luminal domain becomes unfavorable (S10 Fig, S4 Table). However, the overall van der Waals forces become more stabilizing.

We also examined the interactions of the loops with solvent by analyzing the number of contacts and hydrogen bonds formed between all loops and water molecules (Fig 6, S6 Fig, S5 Table). We found that both numbers slightly increase while moving from S2 to S5. As anticipated, the loops were the least hydrated in states S2 and S3 (with bound Cbl) and the most hydrated in state S5 (with Cbl fully permeated). However, note that the hydration of the loops is comparable in states S1, S3, and S4. This implies that during Cbl transport the loops reduce their contacts with water molecules by interacting with other loops (as in S3–S5), luminal domain (S1, S3–S5) or Cbl (S2–S3), which makes sense because the loops contain many aromatic amino acids, such as Tyr, and overall are hydrophobic.

### Enhanced sampling of the BtuB loops affects the energetics of vitamin B_12_ permeation

**Fig 7:**
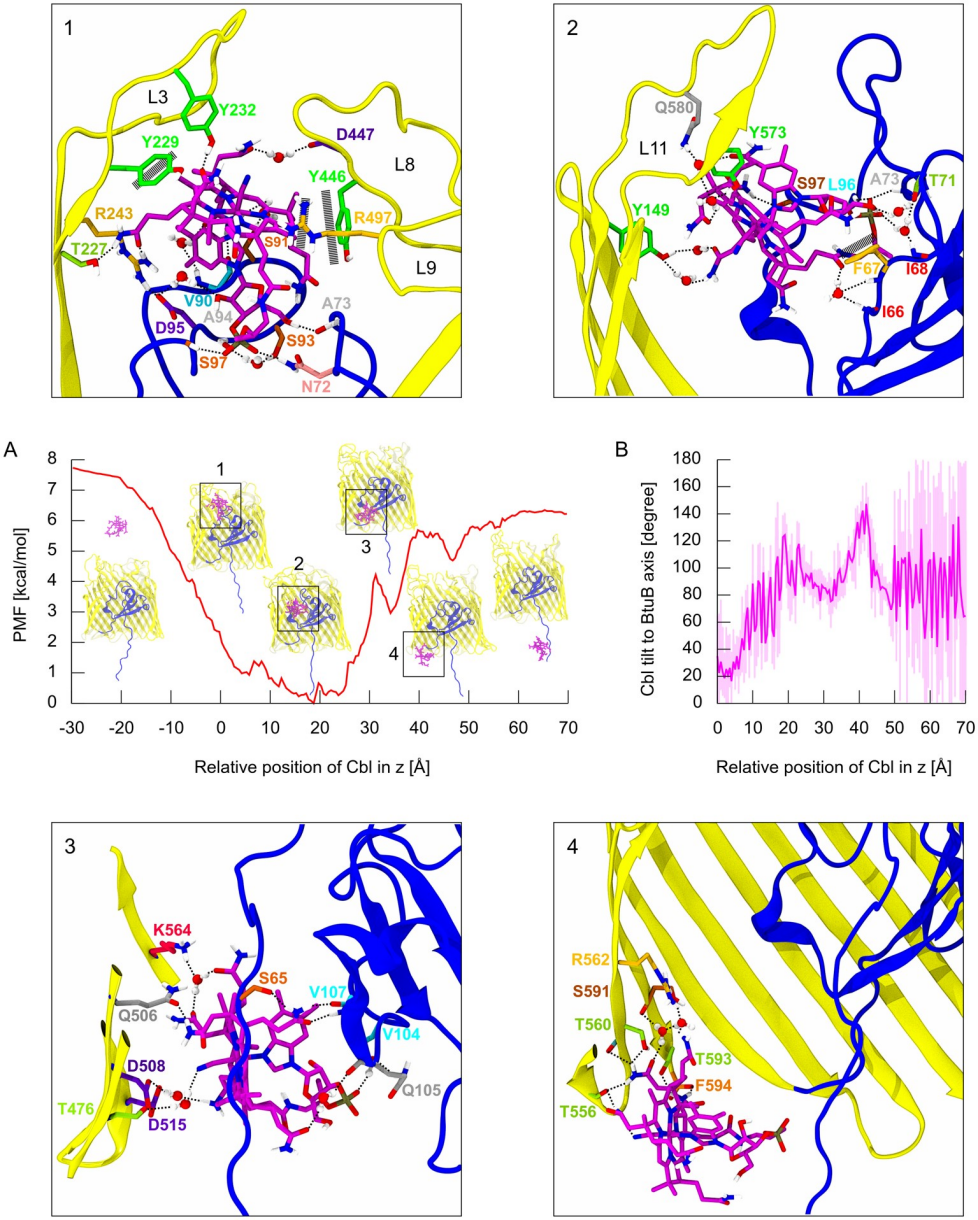
A: The PMF of the association and permeation of vitamin B_12_ through BtuB. B: Rotation of Cbl relative to BtuB during GF-SA-US simulations of permeation. Insets **1**-**4** show representative Cbl binding modes in local energy minima on the transport pathway. The barrel domain is in yellow, luminal domain in blue, and Cbl in magenta. Direct and water-mediated hydrogen bonds are schematically marked by black dotted lines. Stacking interactions are marked by black long-dashed lines. For the axes and coordinate system see Fig 3.

The GF-SA simulations suggested that inadequate probing of the conformational space of BtuB extracellular loops in US could be the reason for the highly unstable PMF of Cbl permeation (S2 Fig). Therefore, we coupled US with the GF-SA approach (see Methods). For each window, we carried out 10 consecutive cycles of GF-SA conjugated with US. Each cycle consisted of 7 ns of GF-SA followed by 3 ns of conventional US. So, in aggregate, 30 ns of US with enhanced sampling of the loops for each window were used to compute the final PMF. With this GF-SA-US approach, the PMF stabilized after 7 cycles (S11 Fig). The PMF of Cbl association with BtuB (z < 0) and its permeation through BtuB (z > 0), together with representative structures corresponding to local energy minima, are presented in Fig 7.

**Fig 8:**
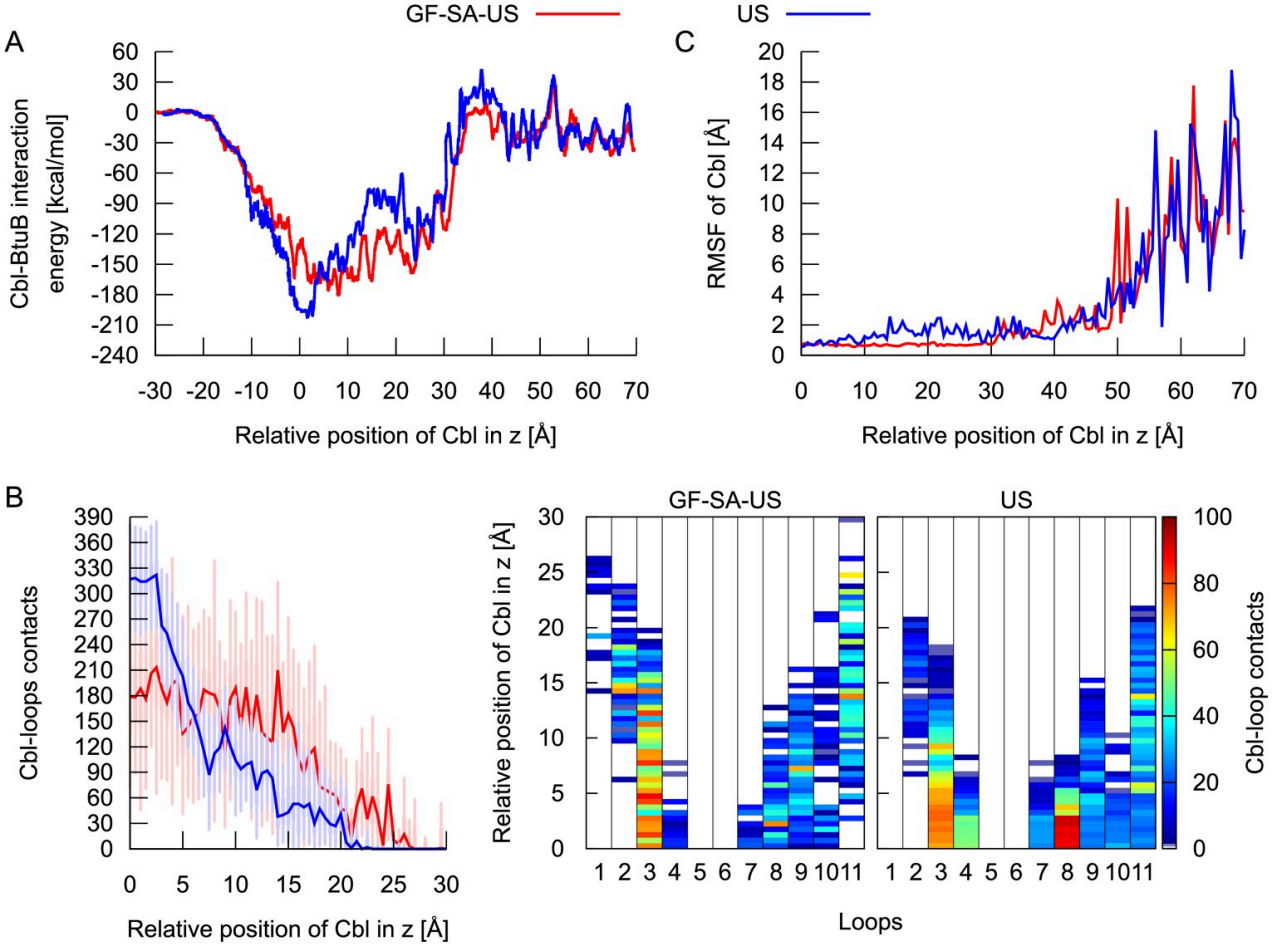
Comparison between various measures obtained from GF-SA-US and US simulations. The interaction energy between Cbl and BtuB (**A**), Cbl contacts with BtuB extracellular loops (**B**), and atomic fluctuations of Cbl (**C**) as a function of the relative position of Cbl (in z) on the transport pathway calculated from the GF-SA-US and US simulations.

On the Cbl association pathway, there are no local energy minima; for z < −20 Å, the PMF stabilizes at about 7.7 kcal/mol. Upon further Cbl movement, up to z = 4 Å, the PMF falls to ca. 1 kcal/mol. This almost linear drop in PMF correlates with the decrease in the interaction energy between Cbl and BtuB (Fig 8A).

**Fig 9:**
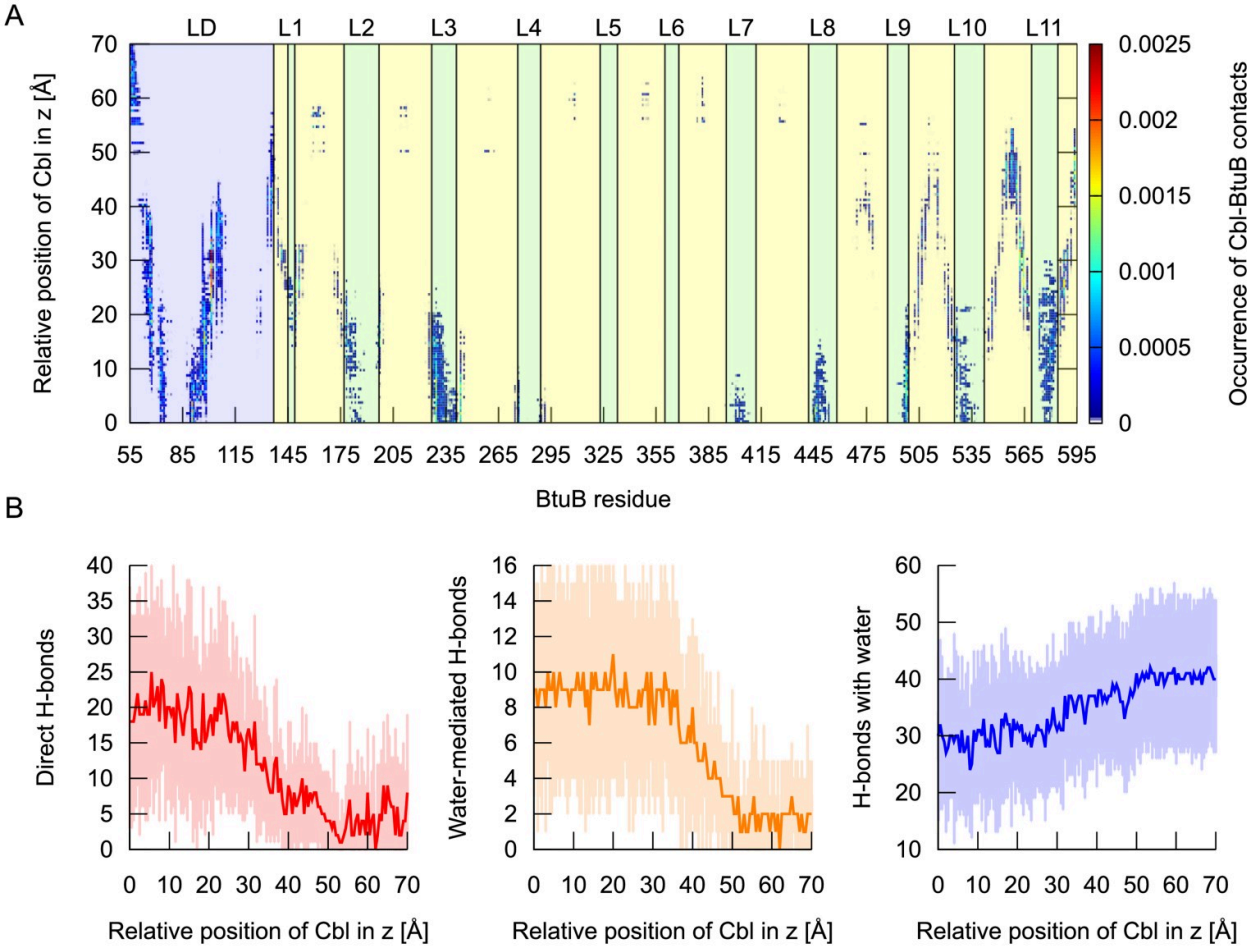
Contacts and hydrogen bonds during vitamin B_12_ permeation through BtuB. The occurrence of contacts between the BtuB residues and vitamin B_12_ as a function of the relative z-position of Cbl (**A**). The number of direct and water-mediated hydrogen bonds formed between Cbl and BtuB, as well as hydrogen bonds created between vitamin B_12_ and water molecules (**B**), during Cbl transport through BtuB.

In z between 4 and 24 Å, the PMF does not exceed 1.4 kcal/mol with the lowest-energy state at z equal 19 Å (Fig 7A). Other multiple energy minima, observed in this region, separated by low barriers might result from imperfect sampling of the degrees of freedom orthogonal to the reaction coordinate, such as the Cbl orientation or loop conformations. Similarly as in SMD simulations, we observed the rotation of vitamin B_12_ relative to the main axis of BtuB by about 120 degrees (Fig 7B). Overall, in this z-region, vitamin B_12_ forms many contacts with the BtuB protein (Fig 9A). Specifically, Cbl interacts with the luminal domain residues 65–67, 72–74, 90–98, and 101, and Cbl interactions with I66, F67, N72, A73, D95, L96, S97 and Q98 occur in almost entire z-range of 4–24 Å (Fig 7).

Considering the BtuB loops, Cbl interacts most favorably with L3 and L11 (Fig 9A). The L3 contacts with Cbl are present up to z = 20 Å and become less abundant as z increases. The most frequent Cbl interactions are with L3 residues T227, N228, Y229, D230, A231, Y232, Y233 and R243 (Fig 7). Most interactions between Cbl and L11 occur for z = 10–18 Å, but some (with L11 residues Y573, Y577, Y579, Q580, and T581) are kept up to z = 30 Å (Fig 7). Cbl interactions with L2 are present up to z = 25 Å, but they are most frequent in the region of z = 10–18 Å. Notably, again, Cbl preferentially interacts with L2 aromatic amino acids, such as Y178, Y183, and F197. Cbl contacts with L8 persist until z over 15 Å and are primarily formed with Y446, D447, D448, and H449 (Fig 7). Up to z = 20 Å, Cbl interacts also with L9 and L10. For L9, these are mainly interactions with R497 and R498 (Fig 7), while for L10—with R526, D528, and Y531. Cbl interactions with L4 and L7 appear up to z = 10 Å. For L7, the contacts are typically formed with Q400, Y402, and F404, while for L4 with D274 and N276. In the z-range of 15–30 Å, Cbl persistently interacts with Y149 (Fig 7), a part of L1. Interestingly, we found no Cbl contacts with L5 and L6 in the entire transport pathway.

The interactions between Cbl and BtuB barrel are detectable only when z > 15 Å. However, they are substantially less abundant and more transient than those with the luminal domain or loops. The most frequent contacts are observed with BtuB residues K504, Q521, L546, K564, E585, and T587.

In the z-range of 24–31 Å, the PMF increases by about 4 kcal/mol (Fig 7A). This correlates well with the increase in the interaction energy between vitamin B_12_ and BtuB (Fig 8A), which is mainly caused by the loss of contacts with all extracellular loops and weakening of interactions of Cbl with the luminal domain. Meanwhile, Cbl contacts with several BtuB barrel residues, including R562, K564, T587, and S589, increase. Furthermore, the tilt of Cbl with respect to the BtuB main axis lowers from 120 to about 80 degrees. After Cbl moving from the local energy maximum at z = 31 Å, the Cbl rotation angle again starts to rise (Fig 7B).

At z = 34 Å, a local energy minimum appears (3.0 kcal/mol), which may reflect the reinforcement of Cbl interactions with the luminal domain. Especially durable contacts are then formed with S64, S65, I66, F67, I101, V104, Q105, R106, and V107 (Fig 7). Also, the number of contacts with BtuB barrel residues, specifically T476, Q506, D508, D515, and K564 (Fig 7), increases.

At z = 40 Å, the PMF reaches a local maximum of 5.7 kcal/mol, which most probably is associated with Cbl losing most of its hitherto existing interactions with the luminal domain. Only contacts with a small fraction of luminal domain (residues 133–137) are maintained. Then, vitamin B_12_ reaches its maximal tilt relative to BtuB equal about 140 degrees. Next, until z = 47 Å, the angle of Cbl rotation decreases to ca. 80 degrees (Fig 7B).

At z = 47 Å, PMF drops to the local minimum of 4.7 kcal/mol due to the interactions of Cbl with the BtuB barrel residues 552–562 (especially S557, H558, and T560), and 591–593 (especially T593) (Fig 7).

When z > 55 Å, Cbl no longer contacts the BtuB barrel domain, but interacts with the extended fragment of the luminal domain (residues 50–60) exposed to the periplasmic space. The PMF reaches a plateau of ca. 6.3 kcal/mol at z > 60 Å. Since Cbl after leaving the BtuB barrel interacts with the extended luminal domain, the PMF plateau after Cbl permeation is ca. 1.4 kcal/mol lower than the PMF plateau achieved at z about −30 Å (when vitamin B_12_ is in the bulk).

On its transport pathway, vitamin B_12_ interacts with BtuB via direct and water-mediated hydrogen bonds (Fig 9B) formed primarily through the phosphate, ribose 5’-hydroxyl, and amide groups (S12B and S12C Fig). The same groups preferentially form hydrogen bonds with bulk water (S12D Fig). The hydrogen bonds are augmented by non-specific interactions involving mainly aliphatic moieties of acetamide and propionamide chains, as well as cyanide group (S12A Fig). This seems reasonable because during vitamin B_12_ transport Cbl interacts with many amino acids with a hydrophobic side-chain, e.g., Tyr, Ile, Leu, Val, Ala, and Phe.

We believe that the high number of transient and non-specific interactions and water-mediated hydrogen bonds formed between Cbl and BtuB helps lower the friction associated with migration of vitamin B_12_ within the BtuB protein and enables its rotation in the first phase of permeation (z = 0–24 Å) (Fig 7B). Next, the average number of direct and water-mediated hydrogen bonds formed by Cbl with BtuB decreases and the average number of Cbl hydrogen bonds with bulk water increases (Fig 9B), which helps in the movement of Cbl from BtuB to the periplasm.

To explore how enhancing the sampling of the BtuB loops influenced vitamin B_12_ transport, we compared various system properties calculated from the GF-SA-US and conventional US simulations. We observed that in conventional US, in z between 0 and 3 Å, the Cbl–BtuB interaction energy is more favorable (by ca. 40 kcal/mol) as compared to the energy in GF-SA-US (Fig 8A). This happens because in US, in this z-region, more contacts (by ca. 140) are formed between Cbl and BtuB loops, particularly L8, L11, and L4 (Fig 8B). As a consequence, the conformations of extracellular loops obtained from conventional MD simulations, even microsecond long, cause tight binding of Cbl in its initial position (z = 0–3 Å), which hampers vitamin B_12_ migration towards the periplasm. This corroborates with a high peak of the SMD force necessary to push Cbl through BtuB (Fig 3C).

Intriguingly, in the z-range of 3–24 Å, the interaction energy between Cbl and BtuB is more negative in the GF-SA-US simulations than in conventional US. Again, the more favorable Cbl–BtuB interaction energy results from the higher number of contacts between Cbl and BtuB loops, especially L3, L2, L9, L10, and L11, in GF-SA-US (Fig 8B). This further implies that loops optimize their conformations to favorably interact with vitamin B_12_ at the early stage of its permeation through BtuB, which leads to greater stabilization of Cbl, reflected in its low atomic fluctuations (Fig 8C). This stabilization appears important because during this phase of transport, vitamin B_12_ rotates, which is crucial for its permeation.

### Conclusions

We determined how the conformational dynamics of the BtuB receptor mediates the transport of vitamin B_12_ through the *E. coli* outer-membrane. We performed a series of conventional and enhanced sampling MD simulations of the full-atomistic model of BtuB embedded in an asymmetric and heterogeneous lipid bilayer mimicking the outer membrane of the *E. coli* K-12 strain.

Our SMD simulations have shown that the release of Cbl from BtuB is mechanically feasible after the luminal domain unfolds at least 175 Å in the direction perpendicular to the membrane. AFM experiments have shown that the non-covalent connection between TonB and Ton box (the N-terminal fragment of the luminal domain) is viable up to ca. 200 Å of the luminal domain extension [39]. Thus, we assumed that vitamin B_12_ permeates through BtuB at the domain extension range of 175–200 Å. Therefore, to simulate Cbl permeation, we extended and constrained the luminal domain at 197 Å, to provide enough room inside the BtuB barrel to mechanically allow for Cbl transport. Further, in the simulations, we found that in the early stage of permeation, Cbl needs to rotate so its corrin ring becomes perpendicular to the surface of the membrane.

Conventional US simulations provided an unstable PMF of vitamin B_12_ transport because of insufficient probing of the conformational space of BtuB extracellular loops in conventional MD. This suggested that detailed description of loop mobility is essential to properly characterize Cbl permeation. Therefore, to enhance loop sampling, we developed a method named Gaussian force-simulated annealing (GF-SA). Using this approach, we investigated loop mobility in several BtuB states corresponding to different stages in Cbl transport cycle. We found that several loops, especially L2, L3, L7, L8, L10, and L11, participate in low-frequency closing–opening motions of the BtuB barrel, which may be crucial for Cbl transport. In the apo state, the loops span their closed and open conformations equally well. After Cbl binding, the equilibrium shifts toward the open states, and during luminal domain unfolding and subsequent Cbl permeation, the loops (especially L3, L8 and L10) tend to close over the Cbl binding site.

The behavior of BtuB extracellular loops resembles the induced-fit mechanism occurring during ligand–protein binding, in which the protein adapts its conformation in response to ligand binding to yield a high-affinity complex. Such phenomenon has been already observed for other TBDTs such as FecA [73], FhuA [74], FepA [75], and ShuA [76]. In these proteins, extracellular loops perform large conformational motions to fold over the ligand and sequester it at the binding site. For BtuB, we found that the fitting of loops is triggered not only by Cbl binding but also by unfolding of the luminal domain. The former affects mainly the mobility of loops L3 and L8, while the latter largely determines the behavior of L2, L10, and L11.

It appears that the conformational fitting arises primarily from the propensity of the loops, rich in hydrophobic amino acids, to maximize the internal non-specific contacts within the Cbl–BtuB complex. Such loop action stabilizes the Cbl molecule during the initial phase of permeation when it undergoes rotation. This facilitates vitamin B_12_ transport through the outer-membrane BtuB protein and may help exclude Cbl dissociation to the external environment. This could also prevent some unwanted solutes present in the environment, such as antibiotics, from entering BtuB, thus ensuring cell protection.

Coupling of conventional US with GF-SA, which enhanced loop sampling, considerably stabilized the PMF of vitamin B_12_ permeation. We found that Cbl passage is energetically favorable up to ca. 20 Å towards the periplasm. The energy barrier on the Cbl permeation route is only about 6 kcal/mol, which implies that transport is rapid. Besides, many transient interactions and water–mediated hydrogen bonds between Cbl and BtuB assure for smooth motion of vitamin B_12_ within and through the BtuB barrel.

Note that our model of Cbl transport is limited by the constrained position of the luminal domain. Additionally, despite the dramatic effect of enhanced sampling of the loops on the PMF of Cbl transport, we expect that some relevant degrees of freedom, such as the orientation of vitamin B_12_, may require even longer sampling to achieve convergence. However, it would be computationally extremely demanding to perform US simulations varying two to three collective variables (Cbl position, Cbl orientation, and extension of the luminal domain) combined with prolonged enhanced sampling of the loops. Nevertheless, our simulations clearly suggest the involvement of the loops “pushing” Cbl in an induced-fit mechanism. Yet, what we could have not captured is that the extending luminal domain could “pull” Cbl towards the periplasm, which would lower the permeation barrier even more.

## Supporting information

S1 Fig Unfolding of the luminal domain during the constant-velocity SMD simulation. No external force was imposed on Cbl. For clarity, only a fragment of the unfolded luminal domain (LD) is shown. The gray area indicates the window in which the release of vitamin B_12_ may occur, corroborating the AFM data of [39].
(TIF)

S2 Fig The potential of mean force (PMF) and its RMSD of the association and permeation of vitamin B_12_ through BtuB obtained with umbrella sampling (US). Every 0.2 ns, the RMSD of the PMF was computed taking into account accumulative data. The colors in the legend denote the simulation time (in ns) after which the shown PMFs were computed. This means that the PMFs were calculated based on the data collected from 0 to 3.5 ns, from 0 to 7.0 ns, etc., and from 0 to 35 ns.
(TIF)

S3 Fig The distributions of the distances between selected BtuB loops’ residues in conventional MD and in GF-SA-MD simulations. The C*α* positions of the residues are marked in red in the inset showing the BtuB structure.
(TIF)

S4 Fig The root-mean-square fluctuations of the BtuB loops in the PC 1 and PC 2 modes. (TIF)

S5 Fig The distances between the BtuB loops and Cbl binding site in different BtuB states. For the definition of states see Fig 4A and for loop numbering Fig 1. The median, interquartile range, and the lower–upper extremes for each data series are provided.
(TIF)

S6 Fig The hydrogen bonds formed within and between the loops as well as between the loops and water in different BtuB states. For the definition of states see Fig 4A. For each data series the median with interquartile range and lower–upper extremes are shown.
(TIF)

S7 Fig The maps of occurrence of contacts, van der Waals and electrostatic interaction energies between the loops in different states of BtuB. For the definition of states see Fig 4A and for loop numbering Fig 1.
(TIF)

S8 Fig The map of occurrence of contacts between the loops and luminal domain in different BtuB states. For the definition of states see Fig 4A and for loop numbering Fig 1.
(TIF)

S9 Fig The occurrence of contacts between the loops and Cbl in different BtuB states. For the definition of states see Fig 4A and for loop numbering Fig 1.
(TIF)

S10 Fig The van der Waals and electrostatic energies between the loops and luminal domain in different states of BtuB. For the definition of states see Fig 4A and for loop numbering Fig 1.
(TIF)

S11 Fig The potential of mean force (PMF) and its RMSD of the association and permeation of vitamin B_12_ through BtuB upon coupling of the GF-SA method with umbrella sampling (US). Every 0.2 ns, the RMSD of the PMF was computed taking into account accumulative data. The colors in the legend number each GF-SA-US simulation cycle after which the shown PMFs were computed. This means that the PMFs were calculated based on the data collected from 0 to 1^*st*^ cycle, from 0 to 2^*nd*^ cycle, etc., and from 0 to 10^th^ cycle.
(TIF)

S12 Fig The occurrence of interactions of vitamin B_12_ with BtuB and water during its permeation through BtuB. Numbering of Cbl heavy atoms used in A–D is shown in E.
(TIF)

S1 Table The distances between the BtuB loops and Cbl binding site in different BtuB states. The medians (Md) and interquartile ranges (*Q*_1_ − *Q*_3_) for each data series are provided. For the definition of states see Fig 4A and for loop numbering Fig 1.
(TIF)

S2 Table The contacts, direct and water-mediated hydrogen bonds formed within the loops in different BtuB states. The medians (Md) and interquartile ranges (*Q*_1_ − *Q*_3_) for each data series are provided. For the definition of states see Fig 4A and for loop numbering Fig 1.
(TIF)

S3 Table The contacts, direct and water-mediated hydrogen bonds formed between the loops and luminal domain in different BtuB states. The medians (Md) and interquartile ranges (*Q*_1_ − *Q*_3_) for each data series are provided. For the definition of states see Fig 4A and for loop numbering Fig 1.
(TIF)

S4 Table The van der Waals and electrostatic interaction energies between the loops and luminal domain in different states of BtuB. The medians (Md) and interquartile ranges (*Q*_1_ − *Q*_3_) for each data series are provided. For the definition of states see Fig 4A and for loop numbering Fig 1.
(TIF)

S5 Table The contacts and hydrogen bonds created between the loops and water molecules in different states of BtuB. The medians (Md) and interquartile ranges (*Q*_1_ − *Q*_3_) for each data series are provided. For the definition of states see Fig 4A and for loop numbering Fig 1.
(TIF)

S1 Video The SMD simulation of Cbl passage and release while unfolding of the luminal domain. (MP4)

S2 Video The exemplary performance of a GF-SA-MD cycle. (MP4)

S3 Video The enhanced sampling of the extracellular loops in GF-SA-MD simulations of S1-S5 states of BtuB. Only MD phases are shown.
(MP4)

S4 Video The first principal component (PC1) extracted from the combined trajectories of S1–S5 states of BtuB. (MP4)

S5 Video The second principal component (PC2) extracted from the combined trajectories of S1–S5 states of BtuB. (MP4)
