# Correction: Extracellular loops of BtuB facilitate transport of vitamin B_12_ through the outer membrane of *E*. *coli*

**DOI:** 10.1371/journal.pcbi.1009696

**Published:** 2021-12-20

**Authors:** Tomasz Pieńko, Joanna Trylska

In the System building of the Methods section, there is an error in the last sentence of the second paragraph:

In the paragraph "GAFF2" should be replaced by "Lipid17 [49] and GLYCAM06j [Kirschner 2008] force fields" and one new reference should be added.

The corrected text should read:

"Bonded and non-bonded parameters for lipids were determined using Lipid17 [49] and GLYCAM06j [Kirschner2008] force fields with the partial atomic charges calculated according to the RESP procedure [45] using Gaussian 09 [46] and antechamber (AmberTools17)."

The added reference should read:

Kirschner KN, Yongye AB, Tschampel SM, González-Outeiriño J, Daniels CR, Foley BL, et al. GLYCAM06: A generalizable biomolecular force field. Carbohydrates. J Comput Chem. 2008;29(40):622-55. pmid:17849372
