# Supplementary figures and images for: Extracellular loops of BtuB facilitate transport of vitamin B12 through the outer membrane of E. coli
Source: PLoS Comput Biol. 2020 Jul 1;16(7):e1008024. doi: 10.1371/journal.pcbi.1008024 (PMC7360065; doi:10.1371/journal.pcbi.1008024)

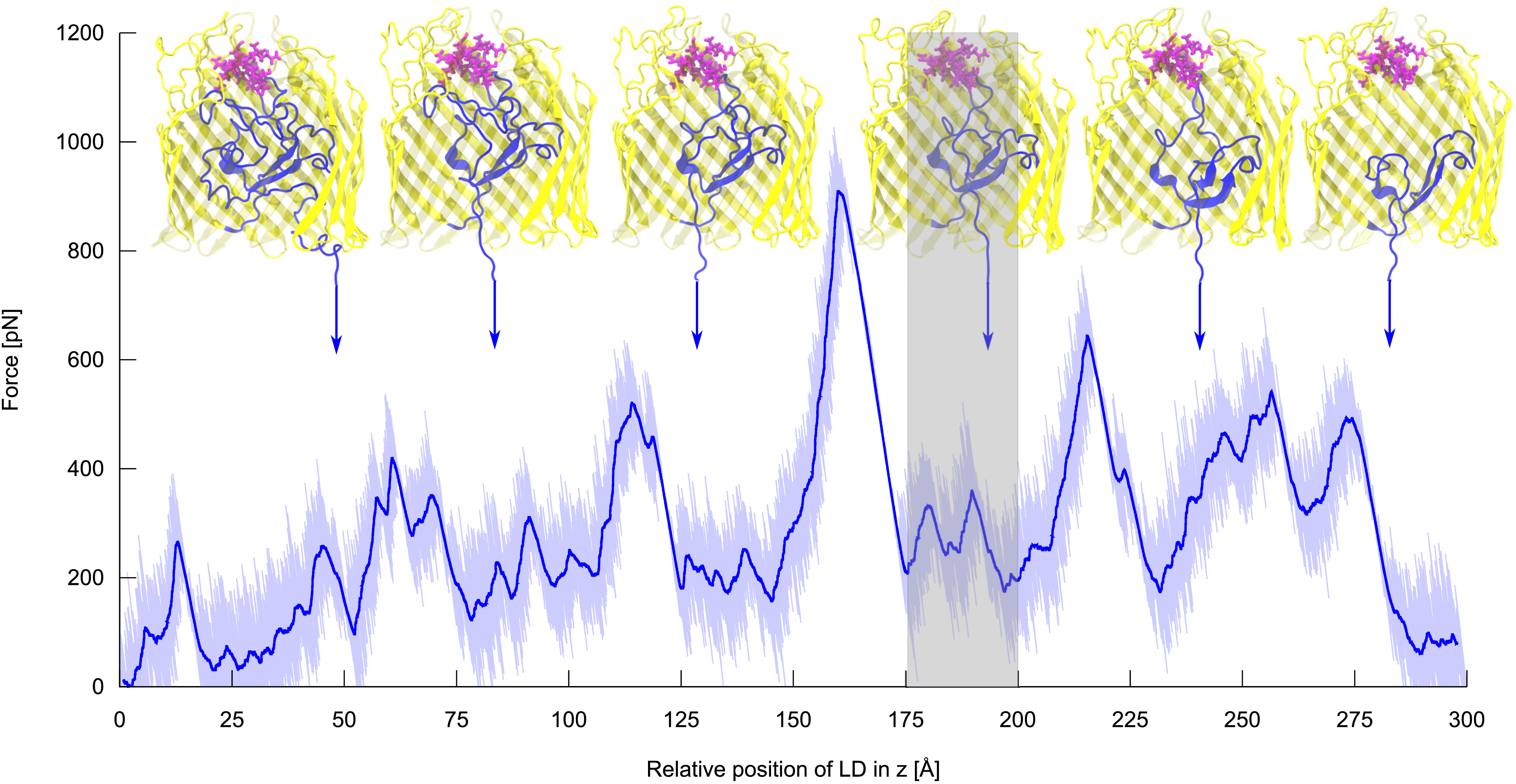

Supplement: S1 Fig — No external force was imposed on Cbl. For clarity, only a fragment of the unfolded luminal domain (LD) is shown. The gray area indicates the window in which the release of vitamin B12 may occur, corroborating the AFM data of [39]. (TIF) [file pcbi.1008024.s001.tif]

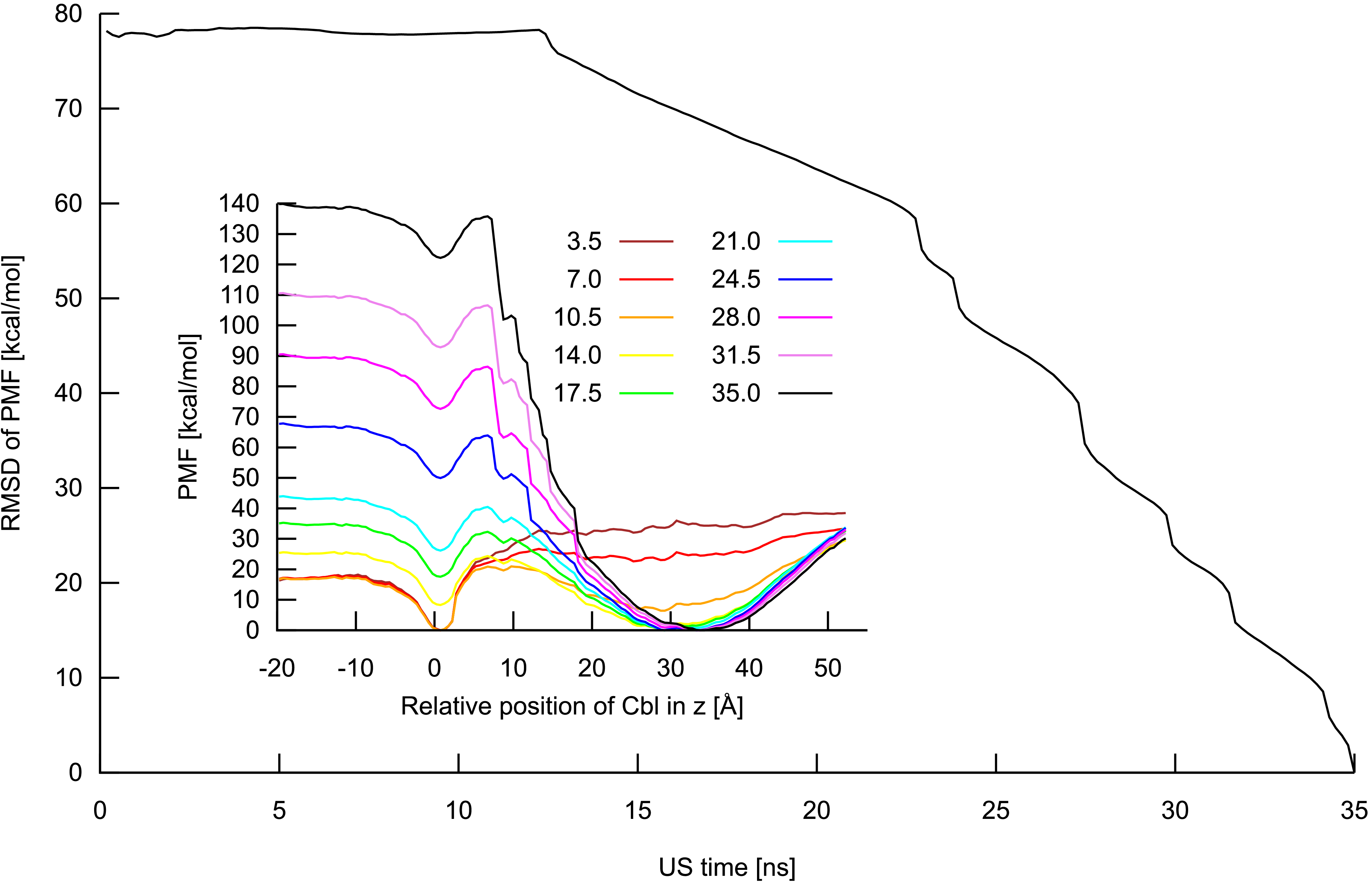

Supplement: S2 Fig — Every 0.2 ns, the RMSD of the PMF was computed taking into account accumulative data. The colors in the legend denote the simulation time (in ns) after which the shown PMFs were computed. This means that the PMFs were calculated based on the data collected from 0 to 3.5 ns, from 0 to 7.0 ns, etc., and from 0 to 35 ns. (TIF) [file pcbi.1008024.s002.tif]

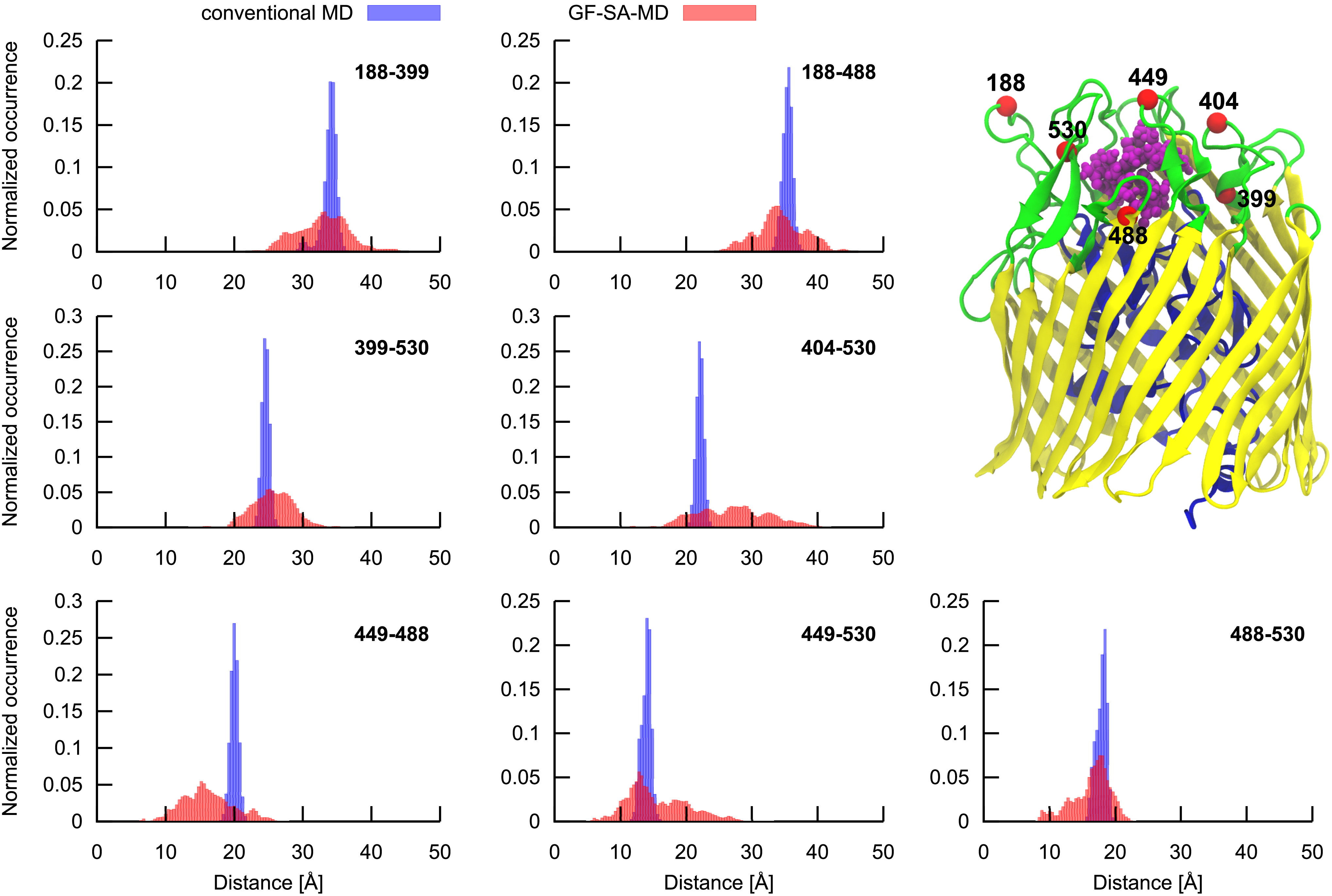

Supplement: S3 Fig — The Cα positions of the residues are marked in red in the inset showing the BtuB structure. (TIF) [file pcbi.1008024.s003.tif]

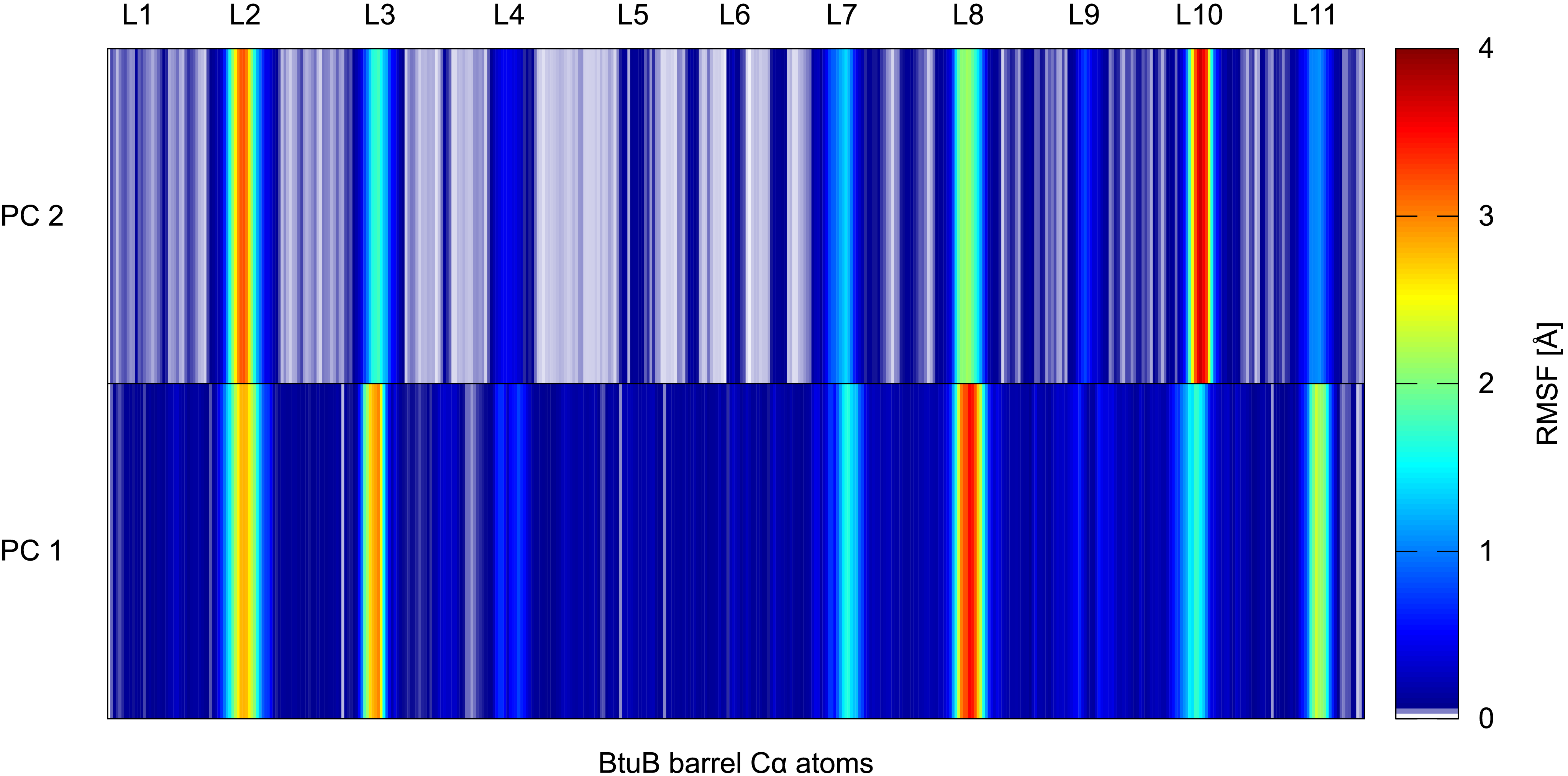

Supplement: S4 Fig — (TIF) [file pcbi.1008024.s004.tif]

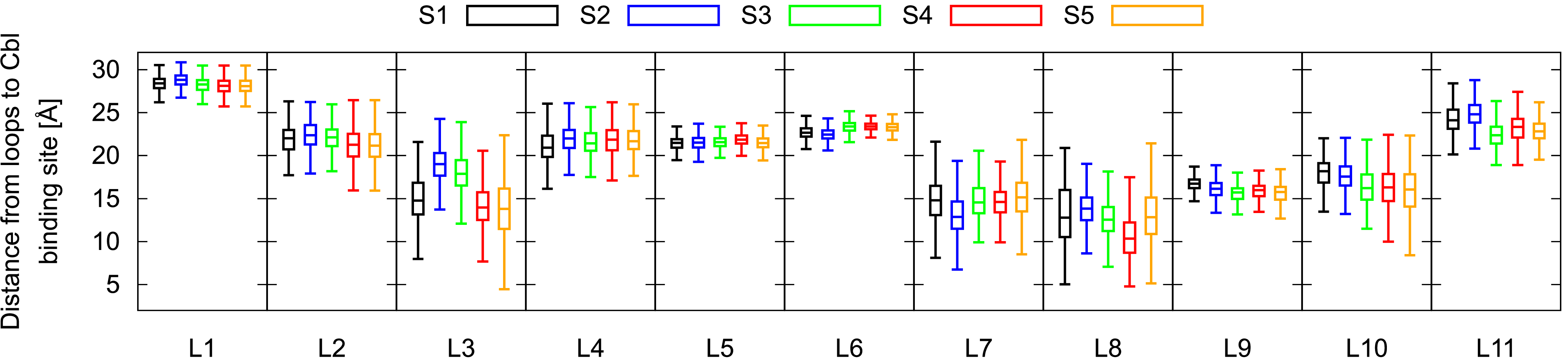

Supplement: S5 Fig — For the definition of states see Fig 4A and for loop numbering Fig 1. The median, interquartile range, and the lower–upper extremes for each data series are provided. (TIF) [file pcbi.1008024.s005.tif]

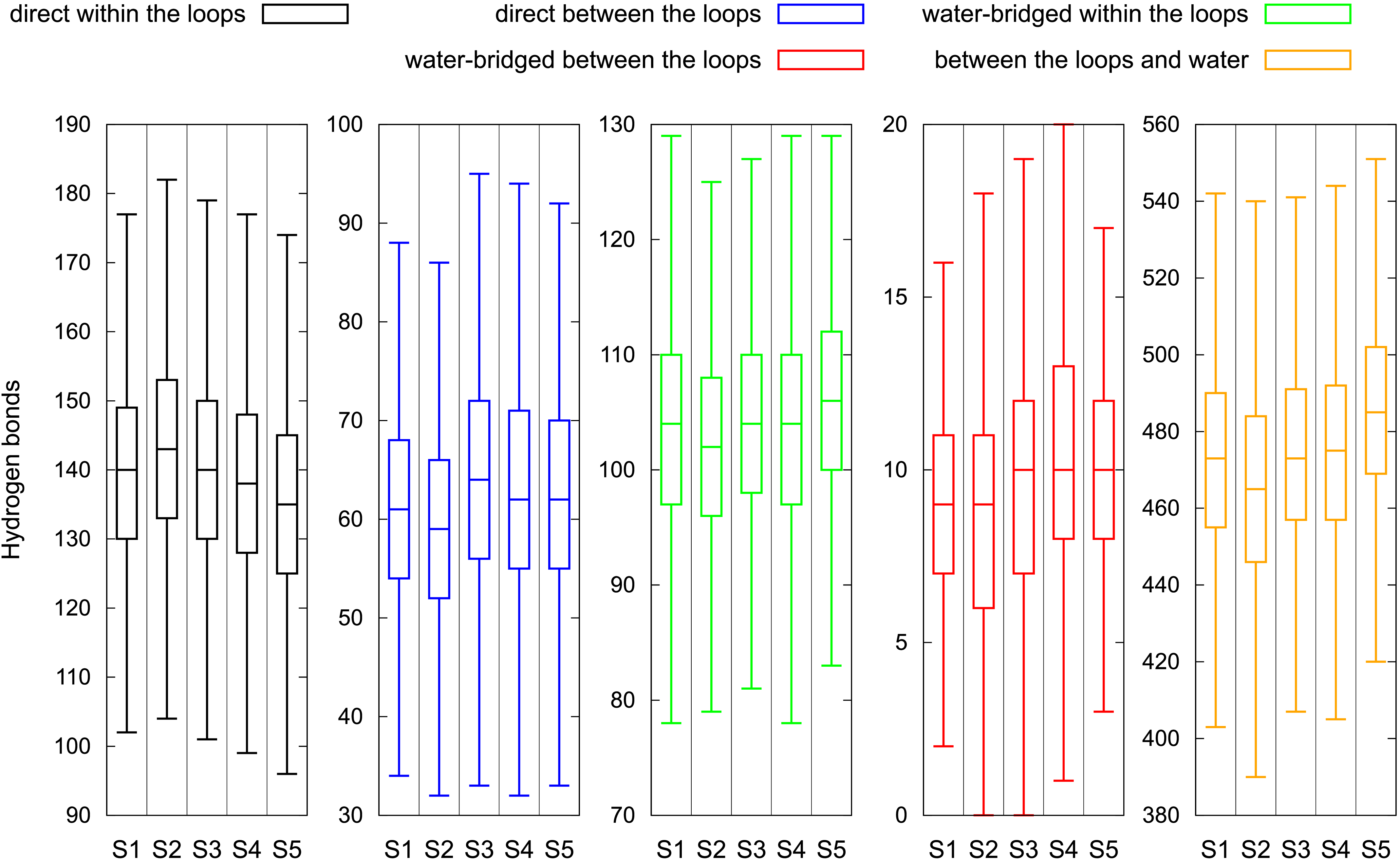

Supplement: S6 Fig — For the definition of states see Fig 4A. For each data series the median with interquartile range and lower–upper extremes are shown. (TIF) [file pcbi.1008024.s006.tif]

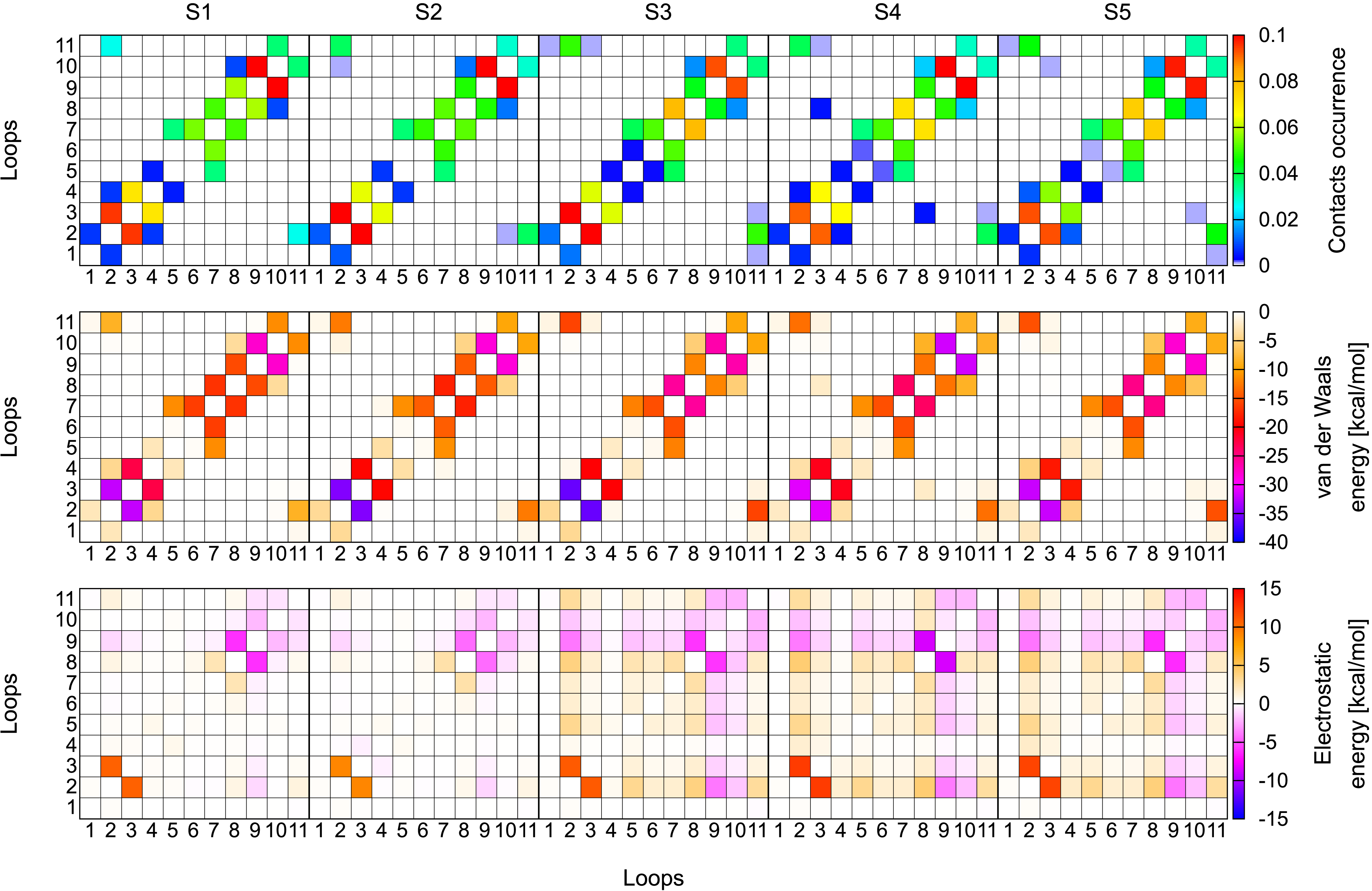

Supplement: S7 Fig — For the definition of states see Fig 4A and for loop numbering Fig 1. (TIF) [file pcbi.1008024.s007.tif]

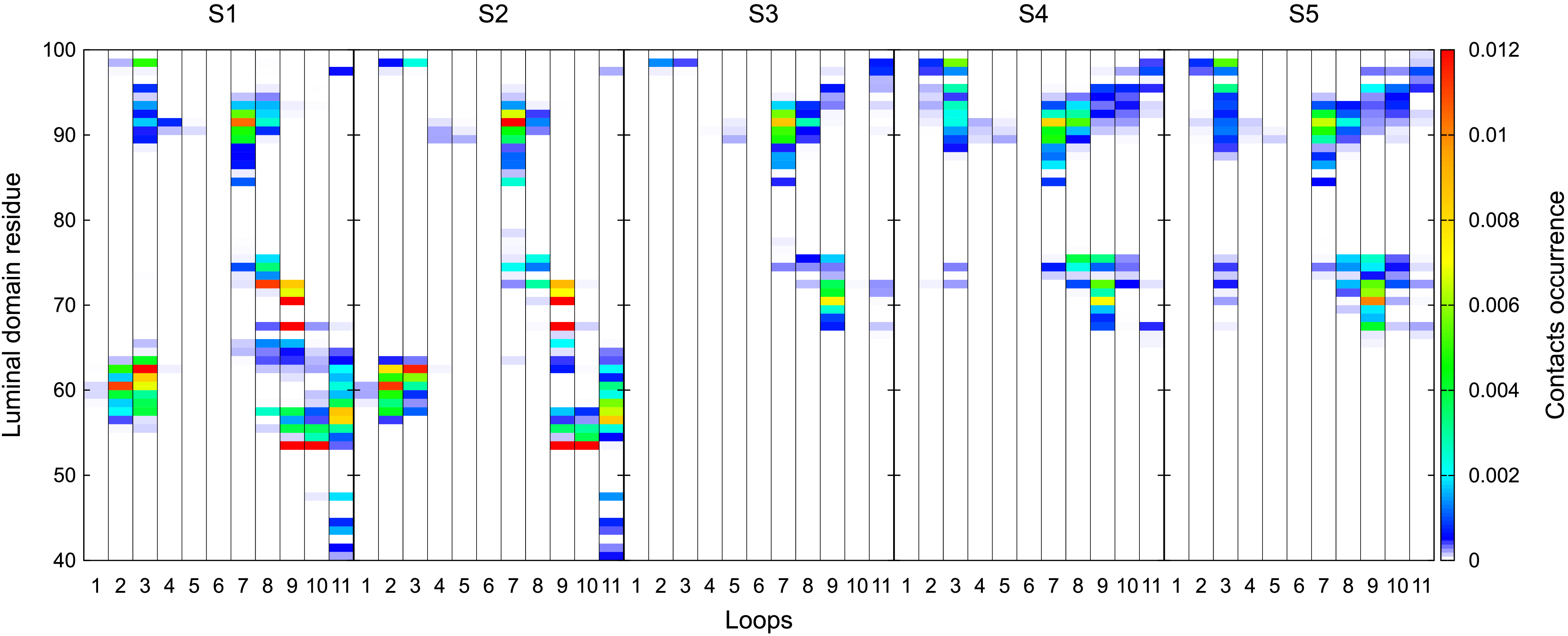

Supplement: S8 Fig — For the definition of states see Fig 4A and for loop numbering Fig 1. (TIF) [file pcbi.1008024.s008.tif]

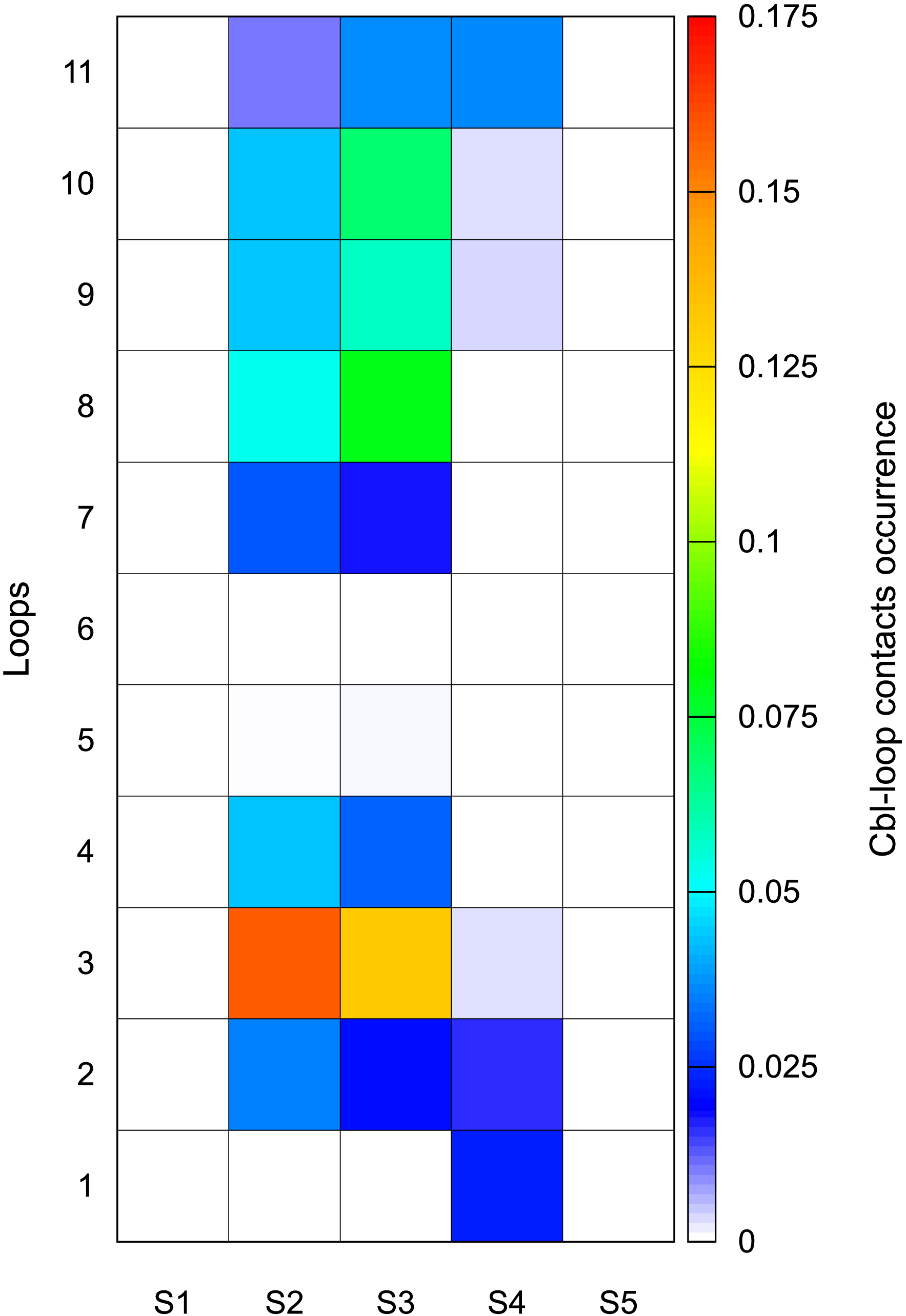

Supplement: S9 Fig — For the definition of states see Fig 4A and for loop numbering Fig 1. (TIF) [file pcbi.1008024.s009.tif]

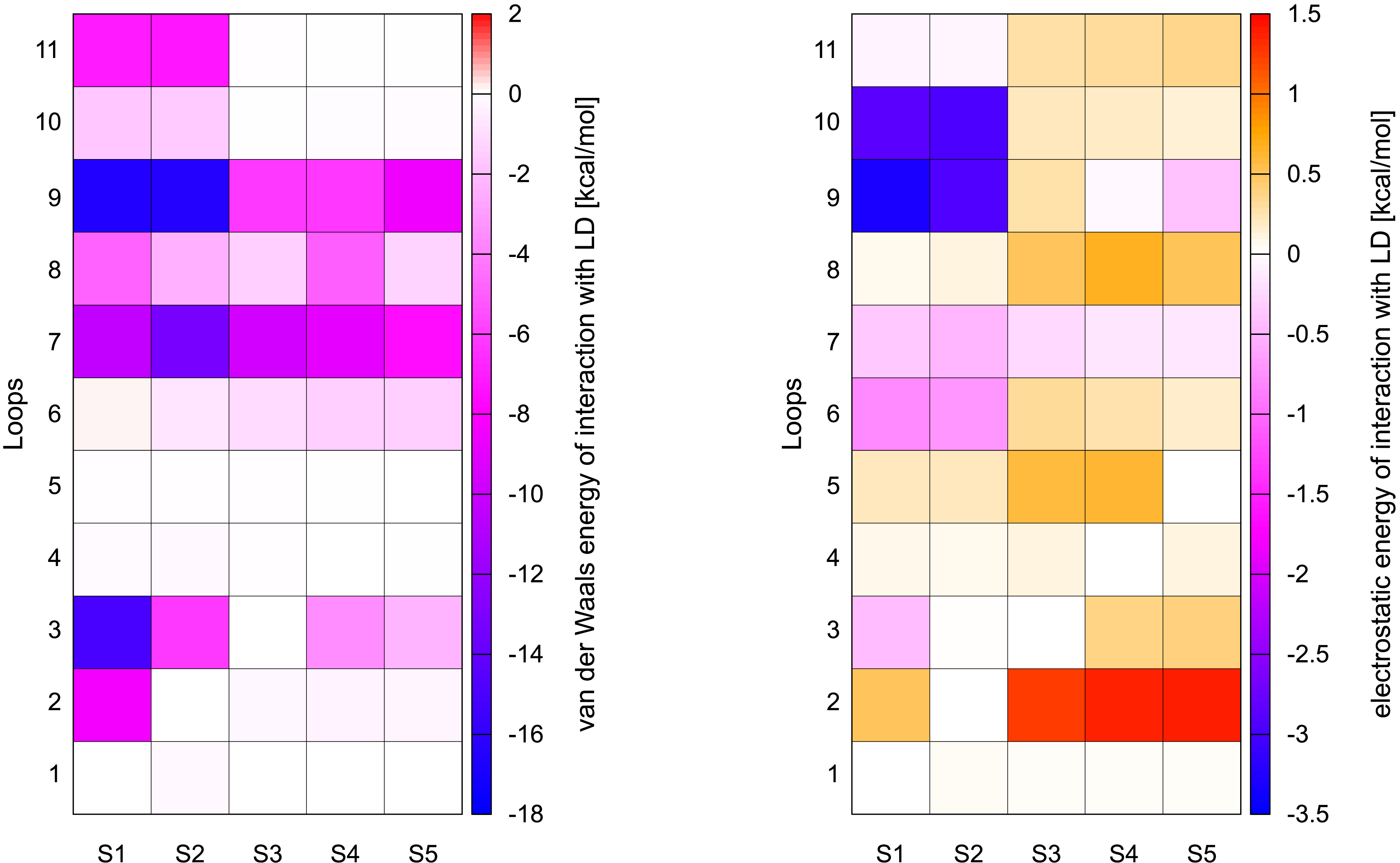

Supplement: S10 Fig — For the definition of states see Fig 4A and for loop numbering Fig 1. (TIF) [file pcbi.1008024.s010.tif]

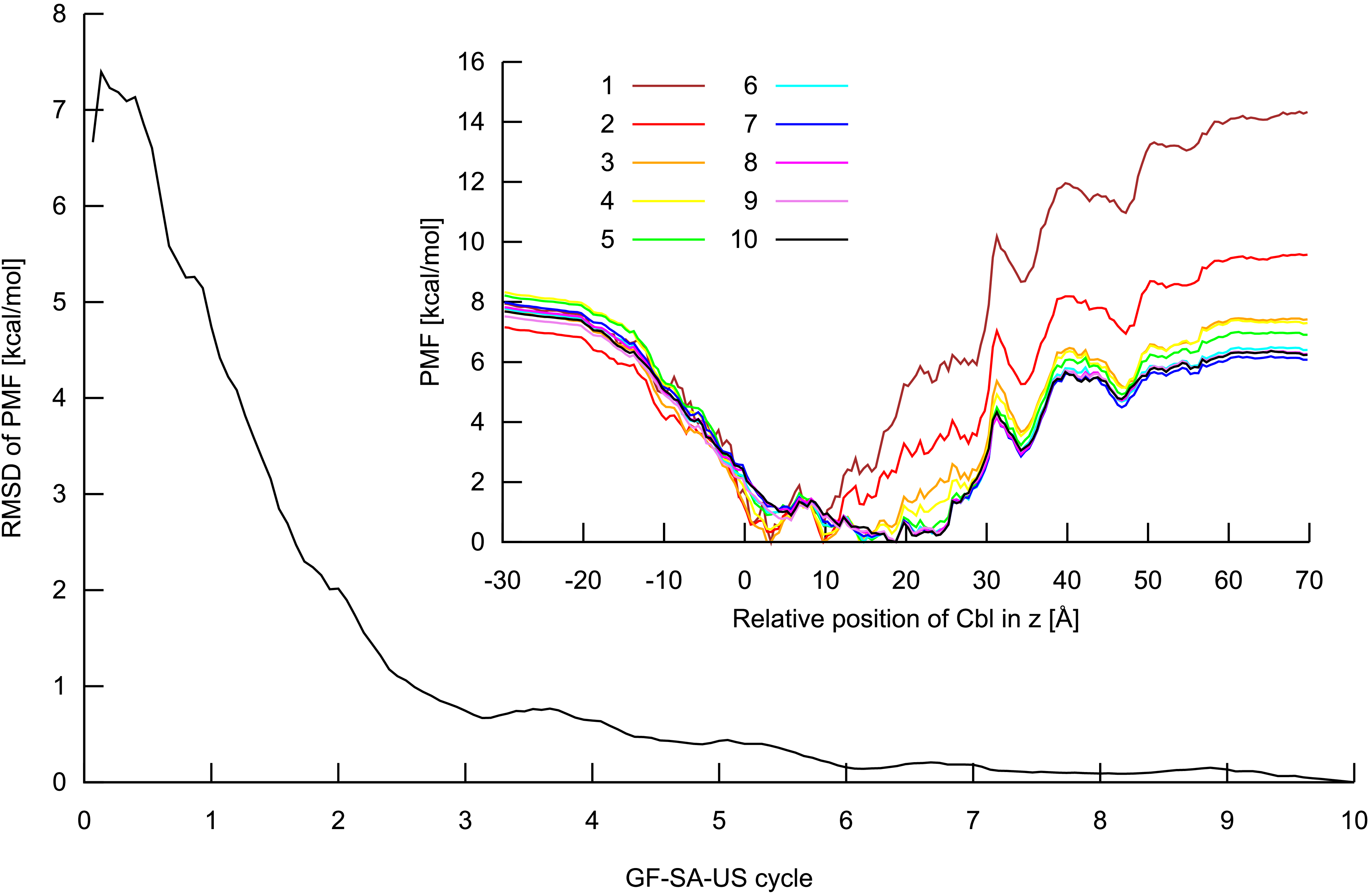

Supplement: S11 Fig — Every 0.2 ns, the RMSD of the PMF was computed taking into account accumulative data. The colors in the legend number each GF-SA-US simulation cycle after which the shown PMFs were computed. This means that the PMFs were calculated based on the data collected from 0 to 1st cycle, from 0 to 2nd cycle, etc., and from 0 to 10th cycle. (TIF) [file pcbi.1008024.s011.tif]

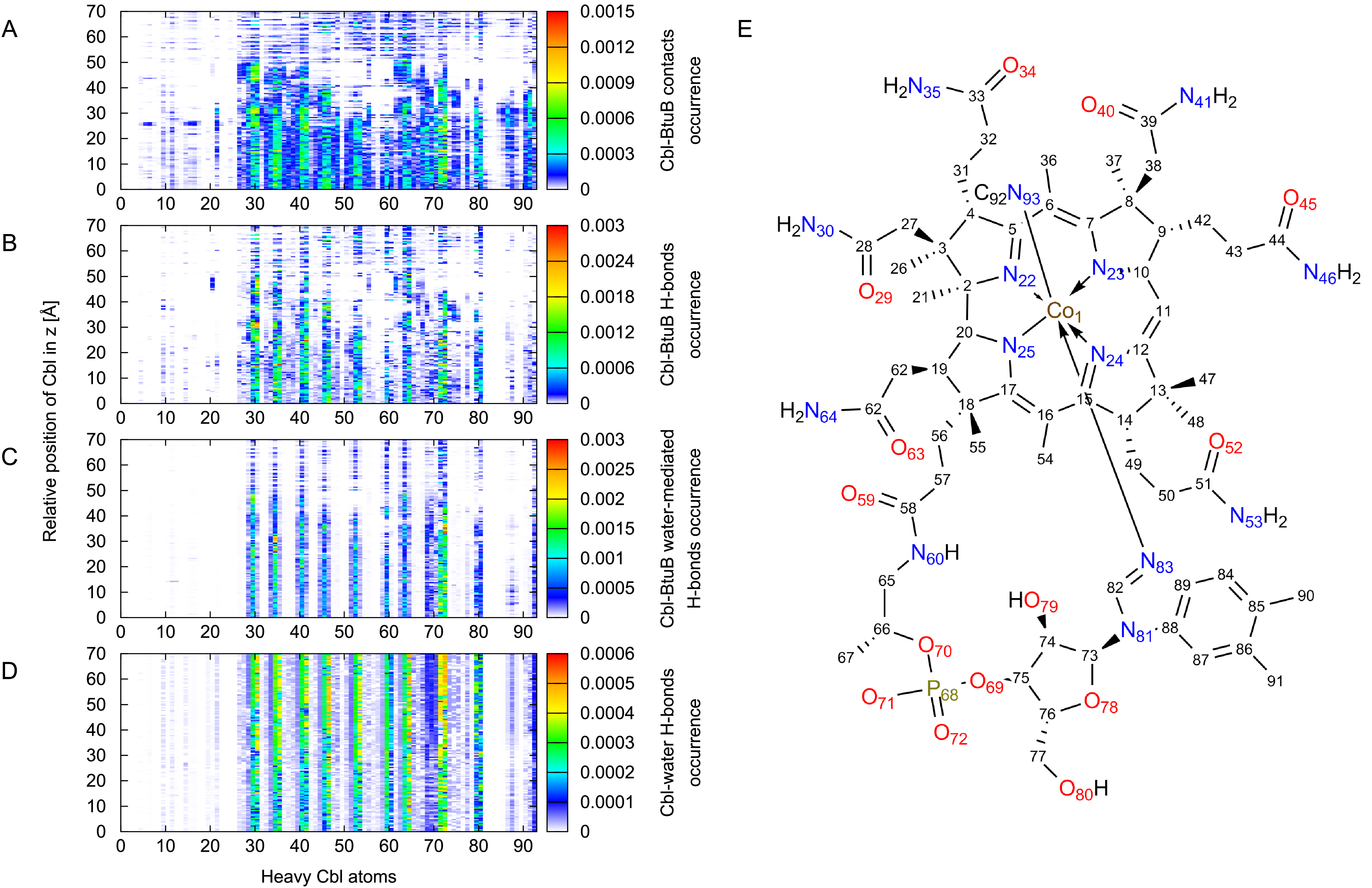

Supplement: S12 Fig — Numbering of Cbl heavy atoms used in A–D is shown in E. (TIF) [file pcbi.1008024.s012.tif]

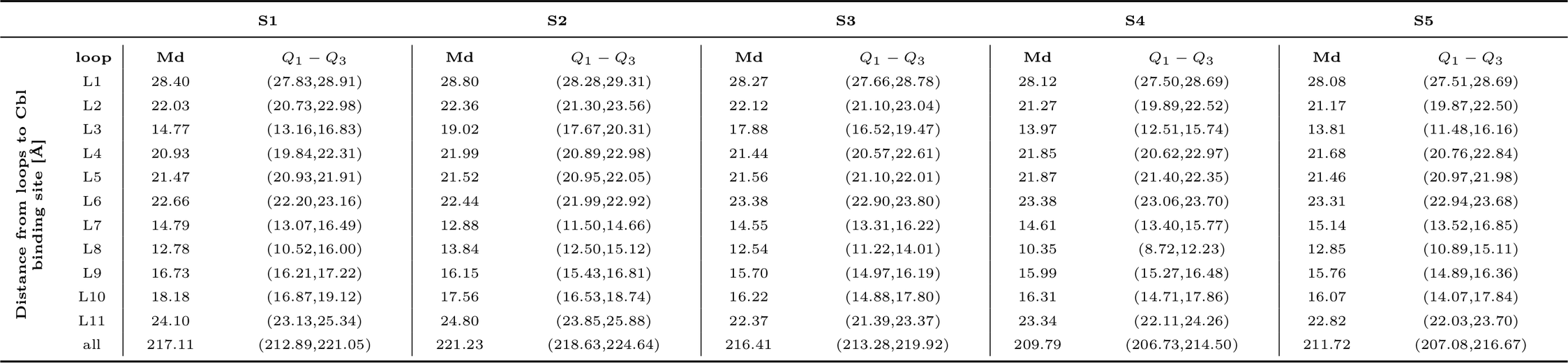

Supplement: S1 Table — The medians (Md) and interquartile ranges (Q1 − Q3) for each data series are provided. For the definition of states see Fig 4A and for loop numbering Fig 1. (TIF) [file pcbi.1008024.s013.tif]

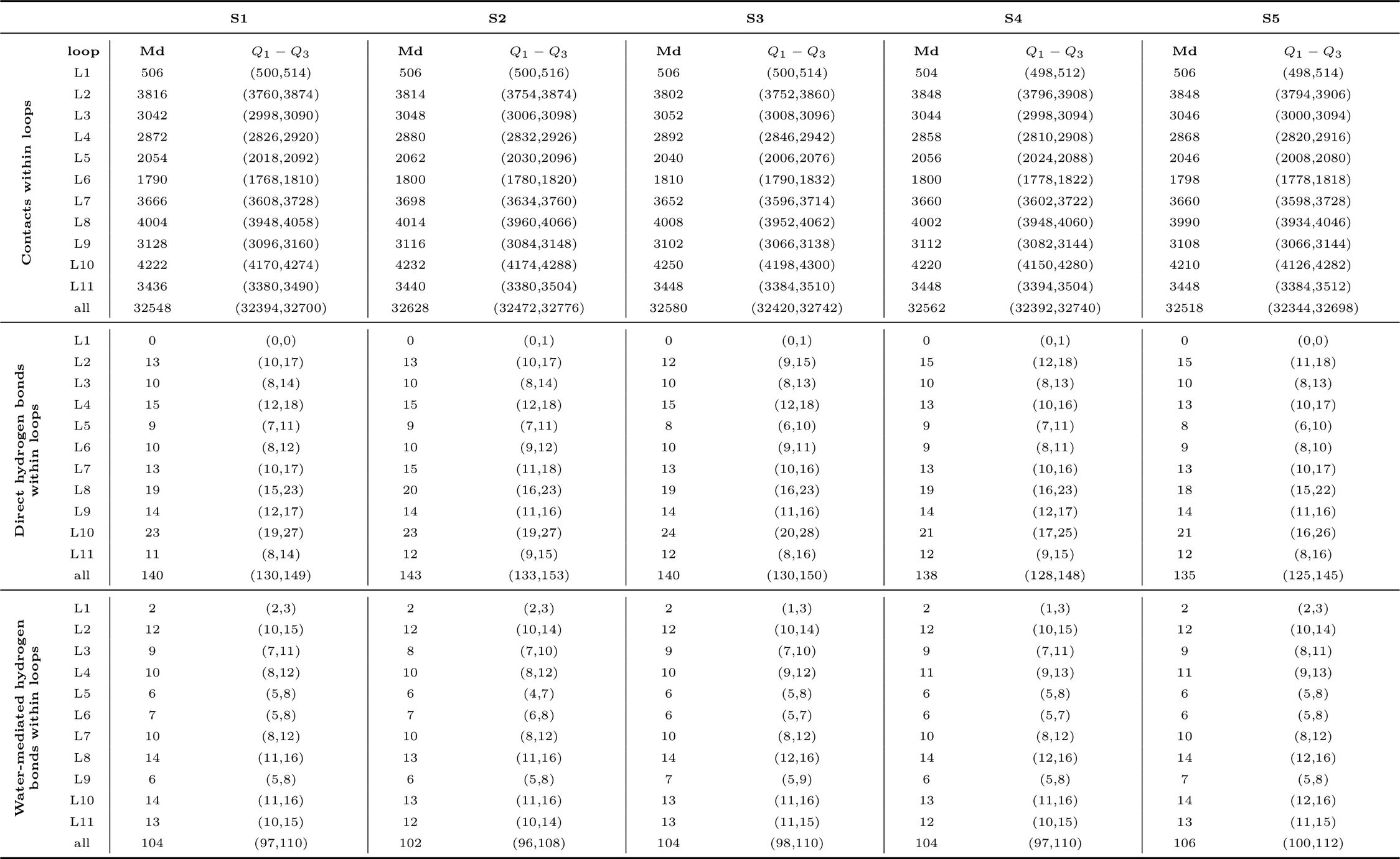

Supplement: S2 Table — The medians (Md) and interquartile ranges (Q1 − Q3) for each data series are provided. For the definition of states see Fig 4A and for loop numbering Fig 1. (TIF) [file pcbi.1008024.s014.tif]

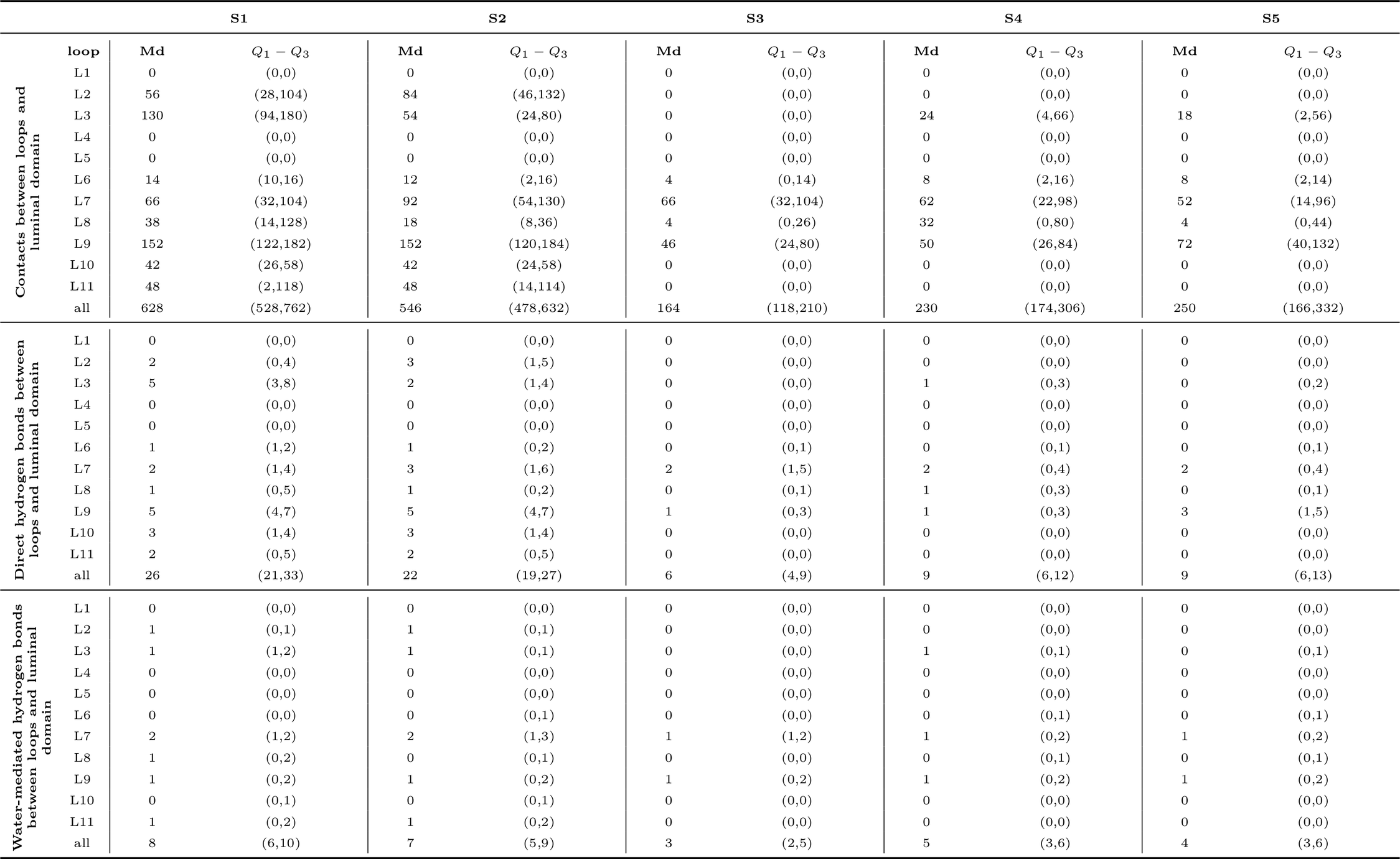

Supplement: S3 Table — The medians (Md) and interquartile ranges (Q1 − Q3) for each data series are provided. For the definition of states see Fig 4A and for loop numbering Fig 1. (TIF) [file pcbi.1008024.s015.tif]

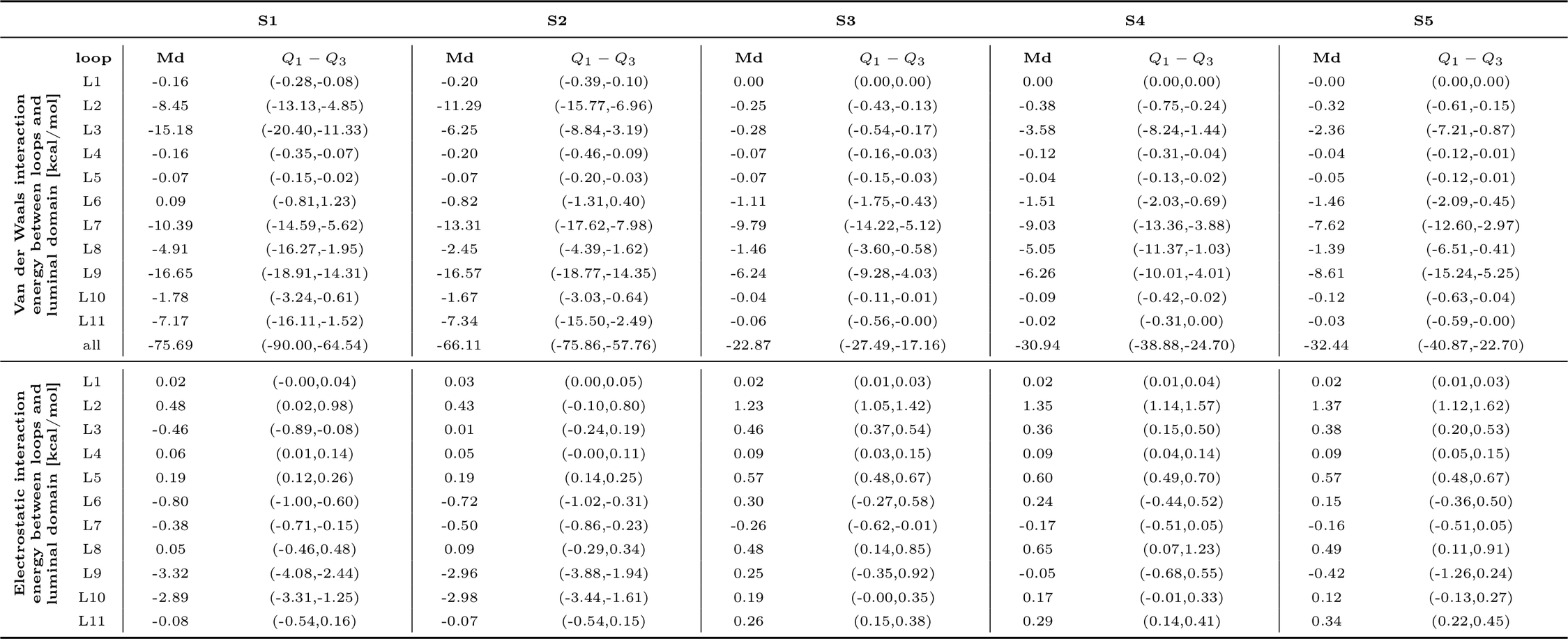

Supplement: S4 Table — The medians (Md) and interquartile ranges (Q1 − Q3) for each data series are provided. For the definition of states see Fig 4A and for loop numbering Fig 1. (TIF) [file pcbi.1008024.s016.tif]

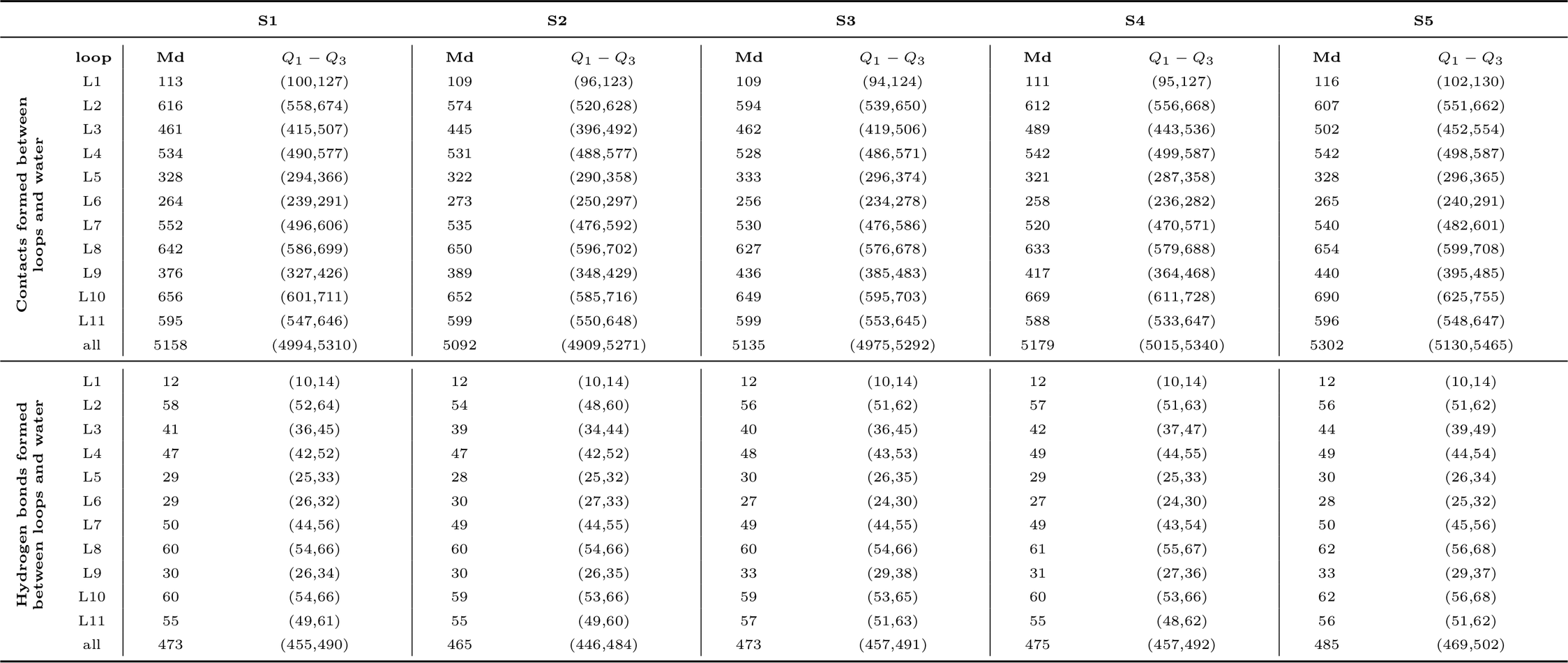

Supplement: S5 Table — The medians (Md) and interquartile ranges (Q1 − Q3) for each data series are provided. For the definition of states see Fig 4A and for loop numbering Fig 1. (TIF) [file pcbi.1008024.s017.tif]
